# Antioxidants and the risk of sleep disorders: results from NHANES and two-sample Mendelian randomization study

**DOI:** 10.3389/fnut.2024.1453064

**Published:** 2024-10-02

**Authors:** Junjie Jiang, Dong Li, Tao Huang, Shan Huang, Hanyu Tan, Zhongfang Xia

**Affiliations:** Department of Otolaryngology, Wuhan Children’s Hospital, Tongji Medical College, Huazhong University of Science and Technology, Wuhan, China

**Keywords:** National Health and Nutrition Examination Survey MR, Mendelian randomization genome-wide association studies BMI, body mass index NCHS, National Center for Health Statistics CDC, Centers for Disease Control and Prevention IRB, Institutional Review Board SNPs, single-nucleotide polymorphisms IVs, instrumental variables LD

## Abstract

**Background:**

Sleep disorders have emerged as a major public health concern. Observational research indicates that antioxidants might mitigate the risk of sleep disturbances, yet the causal relationship remains uncertain.

**Materials and methods:**

This study utilized data from the National Health and Nutrition Examination Survey (NHANES) spanning 2007 to 2018, focusing on adults who reported sleep disorders. The analysis included 25,178 American adults. We examined the association between the Composite Dietary Antioxidant Index (CDAI) and the prevalence of sleep disorders. Additionally, a two-sample Mendelian randomization analysis was conducted to explore the potential causal link between CDAI and the risk of sleep disorders.

**Results:**

Analysis of data from the 2007–2018 NHANES survey revealed a significant negative association between CDAI and sleep disorders (OR = 0.854, 95% CI 0.821–0.888, *P* < 0.001). A multivariable logistic regression model showed that each unit increase in CDAI corresponded to a 14.6% reduction in sleep disorder risk, exhibiting a nonlinear trend where the risk decreased until reaching the inflection point of −0.134. Additionally, MR analysis demonstrated that genetically determined selenium reduces the risk of OSA (OR = 0.992, 95% CI 0.860–0.989, *P* = 0.023). Furthermore, vitamin E (γ-tocopherol) and vitamin C were protective against sleep-wake disorders (OR = 0.016, 95% CI 0.001–0.674, *P* = 0.03) and (OR = 0.049, 95% CI 0.007–0.346, *P* = 0.002), respectively.

**Conclusion:**

Dietary antioxidants may help prevent sleep disorders. However, further studies are required to clarify the pathways through which antioxidants exert this protective effect.

## Introduction

Sleep is a ubiquitous behavior across species, with most people spending about one-third of their day sleeping. It is considered a crucial determinant of human health and performance (1, 2). Sleep disorders are among the most common clinical issues. According to a survey by the World Health Organization, approximately 27% of the world’s population experiences sleep disturbances. The China Sleep Research Society reports that over 300 million Chinese people experience sleep disorders, making China a country with a high rate of sleep problems. The prevalence of sleep disorders is continually increasing due to lifestyle changes, such as night shifts and the frequent use of electronic devices (3–5). Sleep deprivation disrupts physical, psychological, social, and emotional functions, impacting health, safety, and quality of life. Research indicates that individuals suffering from insomnia experience a notably impaired quality of life. The American Sleep Medicine Association and the Society for Sleep Research indicate that sleep disorders are closely associated with cardiovascular diseases, mental health disorders, immune system diseases, cancer, pain, mortality, and overall health (6, 7). However, sleep health remains underrecognized and is a challenging topic in public health. Even after decades of specialized research, the mechanisms regulating human sleep and its functions largely remain elusive. Studies have shown that in animal models, sleep deprivation induces cellular oxidative stress, which can be mitigated by various antioxidants (8, 9). Studies have shown that vitamin C possesses immunomodulatory and antioxidant properties (10, 11). Vitamin E, as a potent antioxidant, can prevent oxidative stress and provides neuroprotective effects for the brain (12, 13). Current research mainly investigates how specific antioxidants impact sleep disorders, but the findings are inconsistent. Notably, meta-analyses have indicated that increasing antioxidant supplements is not always beneficial and can sometimes even increase mortality rates (14).

The Composite Dietary Antioxidant Index (CDAI) is considered an extensive measure for evaluating overall dietary antioxidant capacity. Its calculation is based on six standardized vitamins and minerals, including vitamins A, C, and E, selenium, zinc, and carotenoids derived exclusively from dietary sources (15).

The objective of this research was to evaluate whether CDAI has a preventive effect on the likelihood of sleep disorders. We initially examined data from the National Health and Nutrition Examination Survey (NHANES) to investigate the potential association between CDAI and sleep disorders. Subsequently, we employed the Mendelian randomization (MR) technique, incorporating data from genome-wide association studies (GWAS) on six antioxidants and two subtypes of sleep disturbances, to further assess the causal link between antioxidants and sleep disorder risk. This approach aimed to establish the possible causal impact of antioxidants on the likelihood of developing sleep disturbances.

### Data source

The NHANES survey, which included questionnaires, examination data, and laboratory test results, was conducted with the approval of the National Center for Health Statistics (NCHS) Institutional Review Board. Biennial data releases ensured up-to-date information, and all participants provided written informed consent. The data for these cross-sectional surveys were obtained from the NCHS and are accessible at https://www.cdc.gov/nchs/index.htm. Additionally, we performed a two-sample Mendelian randomization analysis to investigate the causal relationship between dietary antioxidants and different subtypes of sleep disorders.

### Cross-sectional study of NHANES

From 2007 to 2018, the NHANES survey included 50,588 participants. We excluded participants according to specific criteria in our analysis, leading to a final sample size of 25,178 subjects (Supplementary Figure 1). The main outcome measured was the incidence of sleep disorders, defined by responses to the medical conditions questionnaire item: “Over the last two weeks, how often have you been bothered by the following problems: trouble falling asleep or staying asleep, or sleeping too much?” A response of “More than half the days” or “Nearly every day” signified a sleep disorder. On the other hand, answers of “Several days” or “Not at all” showed that no sleep disorder was present.

CDAI was used as the exposure variable, derived from the sum of standardized intakes of six dietary antioxidants (vitamins A, C, and E, selenium, zinc, and carotenoids from food sources). Dietary intake data was obtained through a detailed dietary interview, which assessed the variety and amounts of foods and beverages consumed by respondents in the 24 h before the interview. We also gathered covariates to minimize bias, including demographic factors such as age, sex, ethnicity, level of education, marital status, and household income. Additionally, we documented risk factors associated with sleep disorders, including body mass index (BMI), smoking habits, alcohol use, hypertension, and diabetes. The inclusion and exclusion criteria are detailed in Supplementary Figure 1. The criteria used to diagnose these covariates can be found in Supplementary Table 1.

### Genetic instrumentation and causal assessment

Our methods and findings are reported according to the MR-STROBE checklist (Supplemental File). The research framework and the three fundamental assumptions of Mendelian randomization (MR) are illustrated in Supplementary Figure 2. We utilized data from separate, extensive genome-wide association studies (GWAS) on sleep and circulating antioxidants to evaluate the causal relationships between them. Detailed sources are available in Supplementary Table 2. We used genetic instruments for circulating antioxidants in CDAI, including vitamins A, C, and E (which contains both α-tocopherol and γ-tocopherol), as well as selenium and zinc. Additionally, we used carotene instead of carotenoids, as carotene is the predominant form present in human circulation (16). To identify genetic variants that estimate the causal effects of Vitamins A, C, and E (containing both α-tocopherol and γ-tocopherol), as well as selenium, zinc, and carotene on sleep disorders, we set a significance threshold of *P* < 5 × 10^–6^ to identify single-nucleotide polymorphisms (SNPs) strongly associated with these exposures. We also excluded SNPs with linkage disequilibrium (LD distance > 10,000 kb, r2 < 0.001). Subsequently, we used the PhenoScanner database to examine each SNP and remove those significantly associated with potential confounders and other sleep disorder-related traits. Sleep disorders as outcome variables encompass a range of conditions, including obstructive sleep apnea (OSA), and sleep-wake disorders. To better understand these conditions, we analyzed different subgroups of sleep disorders. To prevent bias, we ensured that the exposure and outcome cohorts had no overlapping samples.

### Statistical analysis

In our NHANES analysis, we utilized multivariable-adjusted logistic regression to investigate the relationship between CDAI and sleep disorders. We evaluated three covariate adjustment models: Model 1 was unadjusted, Model 2 was adjusted for gender, age, and ethnicity, while Model 3 included additional variables.

Due to NHANES’s complex, multi-stage, probability sampling design, this study accounted for weights in the statistical analysis to address unequal selection probabilities. All participants completed the first day’s dietary recall, so the “minimum common denominator” rule was used to determine the “day 1 dietary sample weight.” Since the data covered 12 years across 6 cycles, the weight was calculated as 1/6 * wtdrd 1.

For the two-sample MR analysis, the Inverse-Variance Weighted (IVW) method was primarily used to evaluate the causal link between genetically predicted CDAI and sleep disorders (17). Additionally, four supplementary MR methods—MR Egger, weighted median, weighted mode, and MR-PRESSO—were employed to verify the robustness of the IVW results. Since IVW estimates may be biased due to the inclusion of pleiotropic instruments, sensitivity analyses were performed to address pleiotropy in the causal estimates (18, 19).

## Results

### NHANES cross-sectional analysis

From the 2007–2018 NHANES database, this study enrolled 25,178 individuals. We analyzed the baseline characteristics of those with and without sleep disorders (Table 1). Among these participants, 4,063 reported having sleep disorders. Compared to healthy controls, those with sleep disorders tended to be female, have lower education levels, be White, unmarried, obese (BMI ≥ 30), have lower household income, smoke, drink and have diabetes and hypertension. Participants with sleep disorders had lower CDAI scores than the control group. Intake of vitamins A, C, E, zinc, selenium, and carotenoids was found to have a protective effect against sleep disorders.

**TABLE 1:** Weighted baseline characteristics of participants.

	[Total]	Sleep disorder	No sleep disorder	*P*-value
	*N* = 25,178	*N* = 4,063	*N* = 21,115	
Age:				< 0.001
20–40	8,822 (35.0%)	1,292 (31.8%)	7,530 (35.7%)	
41–60	8,473 (33.7%)	1,493 (36.7%)	6,980 (33.1%)	
61–80	7,883 (31.3%)	1,278 (31.5%)	6,605 (31.3%)	
Gender:				< 0.001
Male	12,413 (49.3%)	1,693 (41.7%)	10,720 (50.8%)	
Female	12,765 (50.7%)	2,370 (58.3%)	10,395 (49.2%)	
Education level				< 0.001
Below high school	5,680 (22.6%)	1,139 (28.0%)	4,541 (21.5%)	
High School or above	19,498 (77.4%)	2,924 (72.0%)	16,574 (78.5%)	
Race/ethnicity				< 0.001
Mexican American	3,691 (14.7%)	487 (12.0%)	3,204 (15.2%)	
Non-Hispanic White	11,120 (44.2%)	1,986 (48.9%)	9,134 (43.3%)	
Non-Hispanic Black	5,123 (20.3%)	815 (20.1%)	4,308 (20.4%)	
Other	5,244 (20.8%)	775 (19.1%)	4,469 (21.2%)	
Married/live with partner				< 0.001
Yes	15,049 (59.8%)	2,060 (50.7%)	12,989 (61.5%)	
No	10,129 (40.2%)	2,003 (49.3%)	8,126 (38.5%)	
BMI:				< 0.001
< 25	7,022 (27.9%)	977 (24.0%)	6,045 (28.6%)	
25–29	8,269 (32.8%)	1,171 (28.8%)	7,098 (33.6%)	
≥ 30	9,887 (39.3%)	1,915 (47.1%)	7,972 (37.8%)	
PIR				< 0.001
Poor	7,901 (31.4%)	1,721 (42.4%)	6,180 (29.3%)	
Not poor	17,277 (68.6%)	2,342 (57.6%)	14,935 (70.7%)	
Drink				0.014
Yes	18,995 (75.5%)	3,128 (77.0%)	15,867 (75.2%)	
No	6,165 (24.5%)	933 (23.0%)	5,232 (24.8%)	
Smoke				< 0.001
Yes	11,358 (45.1%)	2,290 (56.4%)	9,068 (42.9%)	
No	13,820 (54.9%)	1,773 (43.6%)	12,047 (57.1%)	
DM				< 0.001
Yes	4,582 (18.2%)	935 (23.0%)	3,647 (17.3%)	
No	20,596 (81.8%)	3,128 (77.0%)	17,468 (82.7%)	
Hypertension				< 0.001
Yes	10,853 (43.1%)	2,033 (50.0%)	8,820 (41.8%)	
No	14,325 (56.9%)	2,030 (50.0%)	12,295 (58.2%)	
CDAI	0.58 (4.12)	0.28 (4.29)	0.64 (4.08)	< 0.001
Vitamin A, μg	608 (652)	584 (700)	612 (642)	0.019
Vitamin C, mg	82.5 (96.4)	77.1 (97.9)	83.5 (96.0)	< 0.001
Vitamin E, mg	8.29 (6.64)	7.88 (6.83)	8.37 (6.60)	< 0.001
Zinc, mg	11.2 (8.35)	10.7 (8.14)	11.3 (8.38)	< 0.001
Selenium, μg	113 (66.0)	107 (67.6)	114 (65.7)	< 0.001
Carotenoid, μg	9,219 (12,213)	8,450 (12,240)	9,368 (12,203)	< 0.001

Values indicate the weighted mean (SE) or *N* (weighted%). *P*-values are also weighted CDAI, Composite Dietary Antioxidant Index.

In this study, we constructed three weighted logistic regression models to examine the relationship between CDAI and sleep disorders (Table 2). Model 1 indicated that each unit rise in CDAI corresponded to a 14.6% decrease in the risk of sleep disorders (OR = 0.854, 95% CI 0.821–0.888, *P* < 0.001). Model 2, adjusted for age, gender, and ethnicity, showed a 14.3% decrease in sleep disorder risk (OR = 0.857, 95% CI 0.824–0.892, *P* < 0.001). In Model 3, which included additional variables, each unit increase in CDAI was linked to a 10% reduction in sleep disorder risk (OR = 0.900, 95% CI 0.866–0.937, *P* < 0.001).

**TABLE 2:** Association of CDAI and sleep disorder.

	Model 1		Model 2		Model 3	
	OR (95% CI)	*P*	OR (95% CI)	*P*	OR (95% CI)	*P*
CDAI	0.854 (0.821, 0.888)	< 0.001	0.857 (0.824, 0.892)	< 0.001	0.900 (0.866, 0.937)	< 0.001
Q1	Ref		Ref		Ref	
Q2	0.699 (0.615, 0.794)	< 0.001	0.713 (0.627, 0.810)	< 0.001	0.773 (0.677, 0.883)	< 0.001
Q3	0.615 (0.551, 0.688)	< 0.001	0.620 (0.552, 0.696)	< 0.001	0.700 (0.621, 0.788)	< 0.001
Q4	0.634 (0.557, 0.721)	< 0.001	0.644 (0.566, 0.732)	< 0.001	0.742 (0.652, 0.844)	< 0.001
Pfor trend		< 0.001		< 0.001		< 0.001

CDAI, Composite Dietary Antioxidant Index, OR, odds ratio, ref, reference. Model 1, no covariates were adjusted; Model 2, age, sex, and race were adjusted; Model 3, Model 2 plus additional adjustment for the married/live with partner status, education level, poverty income ratios, smoking status, alcohol use, BMI, hypertension, and diabetes were adjusted; *p* < 0.05 was considered statistically significant, 95% CI, 95% confidence interval; OR, odds ratio.

**FIGURE 1:**
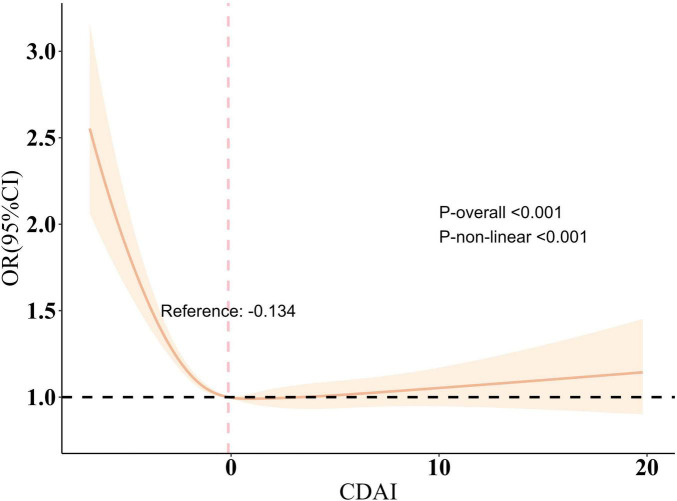
Restricted cubic spline plot of the association between CDAI and sleep disorder. All variables are taken into account and adjusted accordingly. The shaded part represented the 95% CI. CDAI, Composite Dietary Antioxidant Index, OR, odds ratio.

During the sensitivity analysis, CDAI was divided into four categories for trend analysis (Supplementary Table 3). Similarly, the six elements included in CDAI were also divided into four categories (Supplementary Tables 10–15). The results indicated that higher CDAI levels significantly decreased the risk of sleep disorders (*P* < 0.001) (Table 2). Further investigation examined the link between the six dietary components in CDAI and sleep disorders. The findings showed that Vitamins A, C, and E (containing both α-tocopherol and γ-tocopherol), as well as selenium, zinc, and carotene, had a influence on sleep disorders after controlling for confounders (Supplementary Tables 4–9). Additionally, RCS model, adjusted for other variables, revealed a nonlinear relationship between CDAI and sleep disorder risk, indicating a saturation effect (nonlinearity *P* < 0.001) (Figure 1). The inflection point was −0.134; beyond this, each unit increase in CDAI was linked to an 13.1% reduction in sleep disorder risk (OR = 0.869, 95% CI 0.832–0.907, *P* < 0.001). Beyond this point, the association reversed but was not statistically meaningful (OR = 1.008, 95% CI 0.992–1.025, *P* = 0.328).

### MR analysis

**FIGURE 2:**
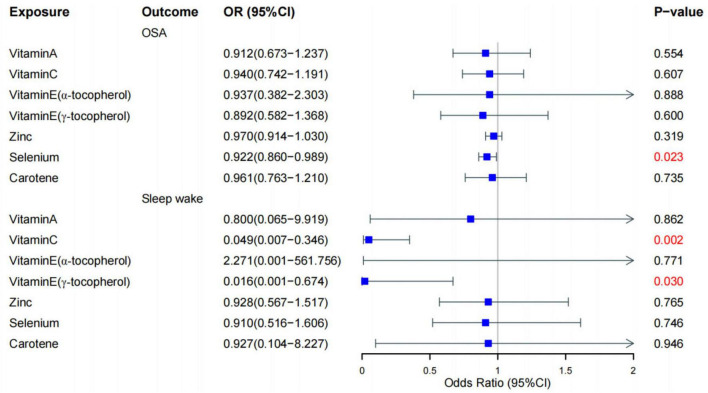
Forest plot for the causal effect of diet-derived antioxidant on the risk of sleep disorder derived from inverse variance weighted (IVW). OR, odds ratio, CI, confidence interval, OSA, obstructive sleep apnea.

**FIGURE 3:**
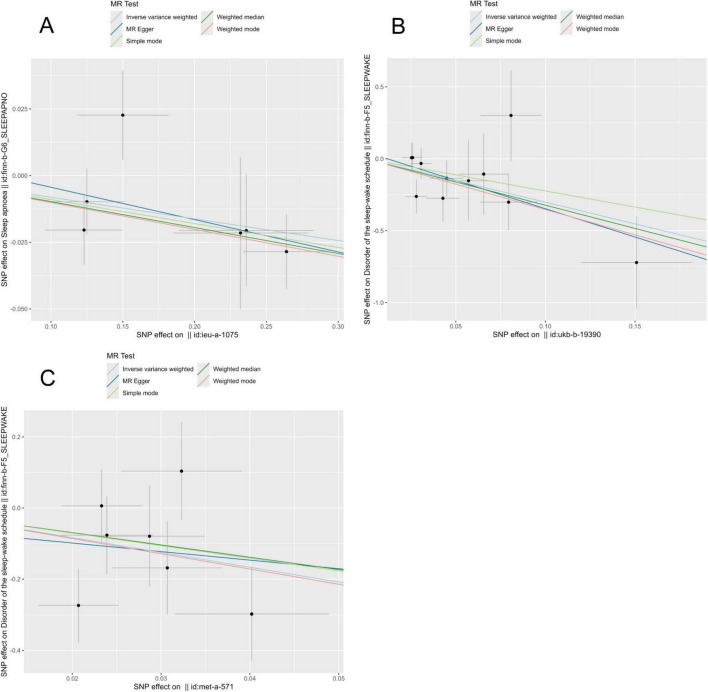
Presents scatter plots from Mendelian randomization (MR) analyses, assessing the impact of various nutrients on sleep-related conditions: **(A)** effect of selenium on obstructive sleep apnea (OSA). **(B)** Effect of vitamin C on sleep-wake. **(C)** Effect of vitamin E (γ-tocopherol) on sleep-wake.

To explore the potential cause-and-effect link between antioxidants and sleep disorders, we conducted an MR analysis. The IV summaries for antioxidants are provided in Supplementary Table 16. The IVW results indicated that selenium (Se) has a protective role in OSA (OR = 0.992, 95% CI 0.860–0.989, *P* = 0.023). Both Vitamin E (γ-tocopherol) and Vitamin C showed positive effects on sleep-wake cycles (OR = 0.016, 95% CI 0.001–0.674, *P* = 0.03) and (OR = 0.049, 95% CI 0.007–0.346, *P* = 0.002), respectively (Figures 2, 3). However, in the other four MR methods, no associations between antioxidants and the likelihood of OSA and sleep-wake cycles were observed (Supplementary Table 17). Subsequently, we conducted a sensitivity analysis of antioxidants on sleep disorders. The findings indicated potential SNP heterogeneity in the MR-Egger analysis of Vitamin E (α-tocopherol) and OSA (*P* = 0.029). No evidence of horizontal pleiotropy was detected (Supplementary Tables 18, 19), and the MR-PRESSO analysis revealed no outliers. Additionally, the leave-one-out analysis showed consistent SNP risk estimates when each SNP was excluded individually (Supplementary Figures 3–5), suggesting the robustness of our results.

## Discussion

According to available literature, this investigation uniquely combines a large-scale cross-sectional and MR method to investigate the link between antioxidant intake and sleep disorder risk. The research delves into the association and potential causality between antioxidant diets and sleep disorders.

Our findings indicate that antioxidant diets may help prevent sleep disorders, providing clinical evidence for the benefits of increased antioxidant intake for sleep health. This study employed data from five NHANES cycles (2007–2018) to explore the relationship linking antioxidant diets with sleep issues. Three models were developed, and the results consistently indicated that higher antioxidant dietary intake was linked to a lower occurrence of sleep disturbances, even after adjusting for various covariates. In the RCS model, this negative association was nonlinear, with a turning point at −0.134.

In our MR analysis, we utilized extensive GWAS datasets to explore the causal links. The findings indicated a causal relationship between selenium and OSA, and between vitamin C and vitamin E with sleep awakenings. However, no significant causal links were identified between other antioxidants and these sleep-related issues. Using the IVW method as the primary analysis, the findings revealed protective effects of genetically determined selenium on OSA, vitamin C and vitamin E on sleep awakenings.

Earlier research has mainly concentrated on the link between individual antioxidants and sleep disorders, with relatively few studies assessing the impact of total dietary antioxidant capacity (DTAC) on sleep disorders. A survey conducted by Chinese researchers identified an increased occurrence of sleep disorders in children with reduced serum vitamin A levels. Similarly, a questionnaire-based study among Japanese students found a link between vitamin A consumption and sleep disturbances (20). Animal experiments by Sei et al. (21) showed that non-rapid eye movement (NREM) sleep significantly improved in vitamin A-deficient (VAD) mice after normal diet restoration, indicating that vitamin A supplementation can enhance sleep quality. Kanagasabai and Ardern (22) found that shorter or very short sleep durations significantly reduced vitamin C levels compared to 7–8 h of adequate sleep. Current studies on how vitamin E affects sleep show inconsistent results. Research by Martens et al. (23) suggested a link between poor sleep health and high serum vitamin E levels. In contrast, Alzoubi et al. (13) found that long-term vitamin E supplementation had protective effects against cognitive impairment caused by chronic sleep deprivation. They also identified vitamin E as a potential molecular target for cognitive impairment due to chronic sleep deprivation. Interestingly, the connection between zinc and sleep exhibits similar trends. A 2009 survey conducted in Jinan, China, assessed serum zinc/copper concentrations and sleep patterns among 890 residents, revealing that adults who slept 7–9 h nightly exhibited the highest serum zinc concentrations (24). Another study in China involving 1,295 children found no notable link between blood zinc levels and sleep quality (25). An Italian randomized, controlled trial discovered that combining zinc with melatonin and magnesium enhanced sleep quality in individuals with primary insomnia (26). A 2021 study revealed that ongoing sleep deprivation impairs both immediate and delayed memory, while selenium supplementation could mitigate this harm (27). Research by Beydoun et al. (28), involving 2,459 adults aged 20–51, found a high prevalence of sleep disorders linked to reduced concentrations of vitamins A, C, E, and carotenoids. This aligns with our study’s findings and further confirms the protective effects of antioxidant diets on sleep disorders.

Our study demonstrates a negative correlation between higher dietary antioxidant intake and a lower incidence of sleep disorders. This association suggests that increasing the intake of antioxidants in daily diets may help prevent or reduce the occurrence of sleep disorders. This preventive effect can be understood as dietary antioxidants alleviating physiological and psychological stress related to sleep disorders through their antioxidant properties and neuroprotective effects, thereby reducing the risk of sleep disorders.

In further analysis, we confirmed a nonlinear relationship between the Composite Dietary Antioxidant Index (CDAI) and sleep disorders. Before the inflection point, each unit increase in CDAI reduced the likelihood of sleep disorders by 11.3%. However, beyond this point, the association reversed and became statistically insignificant (*p*-value > 0.05).

Our research further reveals the potential biological mechanisms by which dietary antioxidants affect sleep disorders. These antioxidants not only directly influence sleep quality through their antioxidant and neuroprotective effects but also play an important role in sleep disorder risk through interactions with genetic variations. Specifically, antioxidants such as vitamins A, C, and E, as well as zinc and selenium, regulate neurotransmitter levels, brain function, and related gene expression through various physiological and biochemical pathways, thereby exerting their effects on sleep regulation. The following sections will discuss these mechanisms in detail and their roles in the prevention of sleep disorders.

Vitamin A and its derivatives (e.g., retinoic acid, RA) regulate sleep through various mechanisms (29). RA influences EEG delta activity during NREM sleep, directly related to sleep demand, intensity, and continuity. RA plays a key role in synaptic plasticity, learning, and memory, impacting hippocampal long-term potentiation (LTP) and depression (LTD), and regulating neurotransmitters like dopamine and serotonin. Lack of sleep greatly influences RA-related gene activity, suggesting that RA signaling plays a role in sleep recovery (30–34). Vitamin C (VC) is crucial for neurotransmitter synthesis and mood regulation, functioning as an essential element for dopamine β-hydroxylase and tryptophan-5-hydroxylase, facilitating the conversion of dopamine to norepinephrine (NE) and tryptophan to 5-hydroxytryptophan (35). Chronic vitamin C deficiency lowers NE and serotonin levels, potentially leading to depression, a major psychological cause of insomnia. Additionally, vitamin C’s antioxidant properties protect the nervous system from oxidative damage. Supplementation benefits stress-related conditions like depression and anxiety, often associated with insomnia (36). As a potent antioxidant, vitamin E influences the hypothalamic orexin system through its α-tocopherol component, activating Nrf2 and triggering antioxidant response elements (ARE), enhancing cellular antioxidant capacity (37, 38). This activation increases hypothalamic orexin expression and turnover, generating orexin-specific peptides, providing neuroprotection and potentially improving sleep quality (39, 40). Zinc’s limited permeability through the blood-brain barrier enables swift entry into the central nervous system, potentially triggering sleep-promoting pathways (41). Zinc functions through the GHS-R-related gene (GPR39), a receptor abundantly found in the amygdala, hippocampus, and auditory cortex, influencing neuronal activity (42, 43). Deficiency in GPR39 is linked to depression-like symptoms and a higher risk of Alzheimer’s disease, both of which are closely connected to sleep disorders (44–47). Selenium, functioning as a cofactor for antioxidant enzymes such as GPx, mitigates memory deficits in sleep-deprived rats by stabilizing oxidative stress in the hippocampus. This is demonstrated by the normalization of antioxidant enzyme functions and oxidative stress indicators (including GSH/GSSG ratio, GPx, catalase, and SOD) following selenium administration (27, 48, 49).

Through Mendelian randomization analysis, we also identified causal relationships between specific antioxidants (such as selenium, vitamin C, and vitamin E) and sleep disorders. This suggests that certain individuals may have a genetically higher demand or sensitivity to these antioxidants. The interaction between these genetic variations and antioxidant intake may influence the risk of sleep disorders. Therefore, this is not merely a simple association between dietary antioxidant intake and sleep disorders; it also involves complex gene-environment interactions, particularly between genetic predispositions and dietary habits.

In summary, the significant negative association observed in our study may reflect both the preventive effect of dietary antioxidants on sleep disorders and the low genetic interaction between antioxidant intake and sleep disorders. This suggests that higher antioxidant intake could reduce the risk of sleep disorders through both direct physiological mechanisms and indirect genetic interactions.

These findings offer greater insight into how antioxidants impact sleep. Additional studies are required to clarify these connections and uncover the underlying biological processes.

### Strengths and limitations

Our study presents several strengths. Firstly, integrating cross-sectional data with Mendelian randomization (MR) analysis diminishes the impact of confounding variables and reduces the chance of reverse causation. Secondly, the extensive sample size helps to minimize sampling bias and enhances the robustness of our findings. Additionally, by dietary intervention, we established a comprehensive index that fully considers the interactions between different antioxidant nutrients. Finally, we analyzed the relationships between three subtypes of sleep disorders and antioxidants in our MR analysis. Nevertheless, our study has its limitations. Firstly, the NHANES study examined various racial groups within the US population, while the MR data is derived from a European population. Further investigation is needed to determine whether the conclusions are applicable to other racial or population groups. Consequently, future research should focus on a more homogenous population, and further research is necessary in larger and more diverse populations. Secondly, NHANES identifies sleep disorders through questionnaire surveys, which may underestimate the prevalence of the disease. Moreover, the antioxidants from dietary sources were estimated based on the 24-h recall prior to the interviews, without dynamic follow-up. Finally, our study did not perform reverse analysis to investigate whether sleep disorders could lead to reduced intake of antioxidants. Although there has been extensive discussion about the connection between antioxidant diets and sleep disturbances, our study did not offer concrete proof to confirm this hypothesis.

## Conclusion

In this study combining NHANES cross-sectional data and MR analysis, dietary antioxidants may have a protective effect against sleep disorders, with selenium being beneficial for OSA, and vitamins C and E being advantageous for sleep awakening. Nevertheless, additional studies are essential to elucidate the mechanisms through which antioxidants prevent sleep disorders. Concurrently, we aim to develop innovative preventive measures and recommendations to reduce the impact of sleep issues on human health.

## Data Availability

The original contributions presented in this study are included in this article/Supplementary material, further inquiries can be directed to the corresponding author.
