# Supplementary material for: Antioxidants and the risk of sleep disorders: results from NHANES and two-sample Mendelian randomization study
Source: Front Nutr. 2024 Oct 2;11:1453064. doi: 10.3389/fnut.2024.1453064 (PMC11480095; doi:10.3389/fnut.2024.1453064)
Supplement: Supplementary file 1 [file Table_1.docx]

**Table S1** Diagnostic criteria of covariates in NHANES

| Variable | Diagnostic criteria |
| --- | --- |
| Age | The oldest age in the population is just 80 years old, and the age of education is required to be greater than or equal to 20  years old, so 20-80 is divided into three age groups, namely 20-40, 41-60, and 61-80. |
| Gender | This variable was subdivided into two groups: female and male. |
| Race | Populations or ethnic groups are categorized into four distinct segments: Mexican Americans, non-Hispanic whites, non  Hispanic blacks, and other races. |
| Marital status: | In categorizing marital status, the amalgamation of marriage and cohabitation into a single category is contrasted with the  distinct classification of being single,divorced, widowed, or separated. |
| Annual household income | The poverty-income ratio was delineated as≥1.3 for non-poverty status and<1.3 for poverty status. Each participant had  three to four consecutive blood pressure levels taken. |
| Education level | Educational attainment is categorized as either incomplete or complete with respect to upper secondary education. |
| Body mass index (BMI) | Obesity was defned as a BMI≥30 according to Centers for Disease Control and Prevention (CDC) guidelines. |
| Smoke status | The assessment of smoking status is based on the question: "Smoked at least 100 cigarettes in life?" Respondents who  answer "yes" are considered smokers. Otherwise, they are considered non-smokers. |
| Alcohol consumption | Alcohol status was ascertained based on participants’ responses to the query, which inquired whether they had  consumed 12 or more alcoholic drinks in the preceding year. Participants who responded afrmatively were  classifed as alcohol users, and conversely for those who answered negatively |
| Diabetes mellitus(DM) | The diagnostic criteria for diabetes are: the doctor told you that you have diabetes, or glycohemoglobin HbA1c (%) greater  than 6.5, or use of diabetes medication or insulin. Participants who responded "yes" were classified as having diabetes. |
| Hypertension | Each participant had three to four consecutive blood pressure levels taken. Systolic (SBP) and diastolic blood pressure  (DBP) values were computed as the average of all extant readings. Hypertension was identified when SBP was ≥140  mmHg and DBP was ≥90 mmHg. |

**Table S2** The GWAS summary information of dietary-derived antioxidants and sleep disorders

| Trait | Build | Year | Author | Sample size | PMID | nsnp | Unit | Population |
| --- | --- | --- | --- | --- | --- | --- | --- | --- |
| Vitamin A | HG19/GRCh37 | 2018 | Ben Elsworth | 62,991 | Public | 9,851,867 | SD | European |
| Vitamin C | HG19/GRCh37 | 2018 | Ben Elsworth | 64,979 | NA | 9,851,867 | SD | European |
| Vitamin E (α-tocopherol) | HG19/GRCh37 | 2014 | Shin | 7,725 | 24816252 | 2,545,636 | log10 units | European |
| Vitamin E (γ-tocopherol) | HG19/GRCh37 | 2014 | Shin | 6,226 | 24816252 | 2,544,979 | log10 units | European |
| Zinc | HG19/GRCh37 | 2013 | Evans | 2,603 | 23720494 | 2,543,610 | SD | European |
| Selenium | HG19/GRCh37 | 2013 | Evans | 2,874 | 23720494 | 2,451,527 | SD | European |
| Carotene | HG19/GRCh37 | 2018 | Ben Elsworth | 64,979 | Public | 9,851,867 | SD | European |
| Sleep apnoea | HG19/GRCh37 | 2021 | NA | 16,761 cases / 201,194 controls | Public | 16,380,465 | NA | European |
| Sleep wake | HG19/GRCh37 | 2021 | NA | 190 cases / 216,164 controls | Public | 16,380,459 | NA | European |

**Table S3** Categorize the CDAI by quartiles

|  | Range | Median | Frequent | Percentage |
| --- | --- | --- | --- | --- |
| Q1 | [-7.159,-1.856] | -3.102 | 7150 | 28.39% |
| Q2 | (-1.856,0.213] | -0.859 | 6470 | 25.71% |
| Q3 | (0.213,2.748] | 1.308 | 5870 | 23.32% |
| Q4 | (2.710,77.599] | 5.023 | 5688 | 22.58% |

**Table S4** Association of vitamin A and sleep disorder.

|  | Model1 | | Model2 | | Model3 | |
| --- | --- | --- | --- | --- | --- | --- |
|  | OR (95% CI) | P | OR (95% CI) | P | OR (95% CI) | P |
| vitamin A | 0.862(0.823,0.904) | <0.001 | 0.868(0.828,0.909) | <0.001 | 0.907(0.867,0.949) | <0.001 |
| Q1 | Ref |  | Ref |  | Ref |  |
| Q2 | 0.863(0.761,0.979) | 0.024 | 0.857(0.754,0.975) | 0.021 | 0.905(0.793,1.034) | 0.147 |
| Q3 | 0.621(0.541,0.712) | <0.001 | 0.619(0.540,0.711) | <0.001 | 0.686(0.596,0.789) | <0.001 |
| Q4 | 0.689(0.598,0.793) | <0.001 | 0.695(0.603,0.802) | <0.001 | 0.800(0.697,0.918) | 0.002 |
| P for trend |  | < 0.001 |  | < 0.001 |  | <0.001 |

**Table S5** Association of vitamin C and sleep disorder.

|  | Model1 | | Model2 | | Model3 | |
| --- | --- | --- | --- | --- | --- | --- |
|  | OR (95% CI) | P | OR (95% CI) | P | OR (95% CI) | P |
| vitamin C | 0.864(0.828,0.903) | <0.001 | 0.868(0.830,0.907) | <0.001 | 0.921(0.879,0.966) | 0.001 |
| Q1 | Ref |  | Ref |  | Ref |  |
| Q2 | 0.736(0.656,0.827) | <0.001 | 0.741(0.657,0.835) | <0.001 | 0.815(0.717,0.926) | 0.002 |
| Q3 | 0.623(0.539,0.720) | <0.001 | 0.619(0.534,0.718) | <0.001 | 0.722(0.617,0.844) | <0.001 |
| Q4 | 0.665(0.584,0.757) | <0.001 | 0.681(0.597,0.778) | <0.001 | 0.804(0.698,0.925) | 0.003 |
| P for trend |  | < 0.001 |  | < 0.001 |  | < 0.001 |

**Table S6** Association of vitamin E and sleep disorder.

|  | Model1 | | Model2 | | Model3 | |
| --- | --- | --- | --- | --- | --- | --- |
|  | OR (95% CI) | P | OR (95% CI) | P | OR (95% CI) | P |
| vitamin E | 0.856(0.820,0.893) | <0.001 | 0.870(0.833,0.908) | <0.001 | 0.911(0.871,0.954) | <0.001 |
| Q1 | Ref |  | Ref |  | Ref |  |
| Q2 | 0.683(0.599,0.779) | <0.001 | 0.690(0.604,0.788) | <0.001 | 0.744(0.651,0.850) | <0.001 |
| Q3 | 0.620(0.545,0.706) | <0.001 | 0.640(0.562,0.728) | <0.001 | 0.708(0.620,0.809) | <0.001 |
| Q4 | 0.632(0.559,0.715) | <0.001 | 0.660(0.583,0.748) | <0.001 | 0.765(0.673,0.870) | <0.001 |
| P for trend |  | < 0.001 |  | < 0.001 |  | <0.001 |

**Table S7** Association of zinc and sleep disorder.

|  | Model1 | | Model2 | | Model3 | |
| --- | --- | --- | --- | --- | --- | --- |
|  | OR (95% CI) | P | OR (95% CI) | P | OR (95% CI) | P |
| zinc | 0.877(0.840,0.915) | <0.001 | 0.909(0.869,0.950) | <0.001 | 0.934(0.892,0.978) | 0.005 |
| Q1 | Ref |  | Ref |  | Ref |  |
| Q2 | 0.846(0.741,0.967) | 0.016 | 0.863(0.755,0.987) | 0.033 | 0.922(0.804,1.058) | 0.253 |
| Q3 | 0.634(0.551,0.730) | <0.001 | 0.671(0.584,0.771) | <0.001 | 0.728(0.632,0.838) | <0.001 |
| Q4 | 0.718(0.630,0.819) | <0.001 | 0.803(0.698,0.924) | 0.003 | 0.871(0.756,1.004) | 0.061 |
| P for trend |  | < 0.001 |  | < 0.001 |  | < 0.001 |

**Table S8** Association of selenium and sleep disorder.

|  | Model1 | | Model2 | | Model3 | |
| --- | --- | --- | --- | --- | --- | --- |
|  | OR (95% CI) | P | OR (95% CI) | P | OR (95% CI) | P |
| selenium | 0.855(0.819,0.892) | <0.001 | 0.884(0.845,0.925) | <0.001 | 0.899(0.859,0.942) | <0.001 |
| Q1 | Ref |  | Ref |  | Ref |  |
| Q2 | 0.764(0.674,0.866) | <0.001 | 0.787(0.693,0.893) | <0.001 | 0.812(0.715,0.922) | 0.002 |
| Q3 | 0.664(0.581,0.760) | <0.001 | 0.704(0.616,0.804) | <0.001 | 0.738(0.643,0.848) | < 0.001 |
| Q4 | 0.630(0.552,0.718) | <0.001 | 0.706(0.614,0.812) | <0.001 | 0.738(0.638,0.852) | < 0.001 |
| P for trend |  | < 0.001 |  | < 0.001 |  | < 0.001 |
|  |  |  |  |  |  |  |

**Table S9** Association of carotenoid and sleep disorder.

|  | Model1 | | Model2 | | Model3 | |
| --- | --- | --- | --- | --- | --- | --- |
|  | OR (95% CI) | P | OR (95% CI) | P | OR (95% CI) | P |
| carotenoid | 0.867(0.829,0.906) | <0.001 | 0.871(0.835,0.909) | <0.001 | 0.908(0.871,0.948) | <0.001 |
| Q1 | Ref |  | Ref |  | Ref |  |
| Q2 | 0.799(0.694,0.920) | 0.002 | 0.802(0.695,0.925) | 0.003 | 0.858(0.741,0.995) | 0.045 |
| Q3 | 0.664(0.579,0.761) | <0.001 | 0.675(0.589,0.774) | <0.001 | 0.741(0.643,0.854) | <0.001 |
| Q4 | 0.669(0.585,0.765) | <0.001 | 0.679(0.594,0.776) | <0.001 | 0.770(0.674,0.879) | <0.001 |
| P for trend |  | < 0.001 |  | < 0.001 |  | < 0.001 |

Table S4-S9:

Model 1: no covariates were adjusted.

Model 2: age, gender, and race were adjusted.

Model 3: Model 2 plus additional adjustment for the married/live with partner status, education level, poverty income ratios,

smoking status, alcohol use, BMI, hypertension, and diabetes were adjusted;

p<0.05 was considered statistically signifcant

95% CI 95% confdence interval, OR odds ratio

**Table S10** Categorize the vitamin A by quartiles

|  | Range | Median | Frequent | Percentage |
| --- | --- | --- | --- | --- |
| Q1 | [0,280] | 162 | 7072 | 28.08% |
| Q2 | (280,507] | 389 | 6460 | 25.65% |
| Q3 | (507,825] | 640 | 6041 | 23.99% |
| Q4 | (825,26549] | 1130 | 5605 | 22.26% |

**Table S11** Categorize the vitamin C by quartiles

|  | Range | Median | Frequent | Percentage |
| --- | --- | --- | --- | --- |
| Q1 | [0,21.3] | 10.3 | 6485 | 25.76% |
| Q2 | (21.3,51.9] | 33.8 | 6177 | 24.53% |
| Q3 | (51.9,111.7] | 77.3 | 6117 | 24.29% |
| Q4 | (111.7,2135.5] | 171.9 | 6399 | 25.41% |

**Table S12** Categorize the vitamin E by quartiles

|  | Range | Median | Frequent | Percentage |
| --- | --- | --- | --- | --- |
| Q1 | [0,4.64] | 3.22 | 7224 | 28.70% |
| Q2 | (4.64,7.25] | 5.86 | 6457 | 25.64% |
| Q3 | (7.25,11.07] | 8.83 | 6070 | 24.11% |
| Q4 | (11.07,158.39] | 14.7 | 5427 | 21.55% |

**Table S13** Categorize the zinc by quartiles

|  | Range | Median | Frequent | Percentage |
| --- | --- | --- | --- | --- |
| Q1 | [0,6.93] | 5.12 | 6994 | 27.77% |
| Q2 | (6.93,10.08] | 8.45 | 6402 | 25.42% |
| Q3 | (10.08,14.49] | 11.95 | 6086 | 24.17% |
| Q4 | (14.49,477.53] | 18.52 | 5696 | 22.64% |

**Table S14** Categorize the selenium by quartiles

|  | Range | Median | Frequent | Percentage |
| --- | --- | --- | --- | --- |
| Q1 | [0,71.9] | 53 | 6727 | 26.72% |
| Q2 | (71.9,103.5] | 87.6 | 6343 | 25.19% |
| Q3 | (103.5,143.2] | 120.6 | 6034 | 23.96% |
| Q4 | (143.2,1195.6] | 180.1 | 6074 | 24.13% |

**Table S15** Categorize the carotenoid by quartiles

|  | Range | Median | Frequent | Percentage |
| --- | --- | --- | --- | --- |
| Q1 | [0,2130] | 892 | 6950 | 27.59% |
| Q2 | (2130,5612] | 3648 | 6335 | 25.16% |
| Q3 | (5612,12697] | 8364 | 6061 | 24.07% |
| Q4 | (12697,377178] | 20787.5 | 5832 | 23.16% |

**Table S16** Genetic instrumental variables for dietary-derived antioxidants

| Trait | Sequence | Chr | SNP | Effect allele | Other allele | Eaf | Beta | SE | *P* |
| --- | --- | --- | --- | --- | --- | --- | --- | --- | --- |
| Vitamin A | 1 | 1 | rs12119164 | G | A | 0.742193 | 0.0295741 | 0.00637063 | 3.40E-06 |
|  | 2 | 1 | rs692790 | C | T | 0.874331 | 0.0404149 | 0.00838208 | 1.40E-06 |
|  | 3 | 2 | rs74977546 | A | G | 0.052896 | -0.063514 | 0.0126784 | 5.50E-07 |
|  | 4 | 3 | rs149577802 | T | C | 0.015116 | -0.108795 | 0.0234115 | 3.40E-06 |
|  | 5 | 10 | rs3213829 | G | T | 0.546482 | 0.025976 | 0.00560984 | 3.60E-06 |
|  | 6 | 14 | rs117669768 | A | G | 0.038236 | 0.0789545 | 0.0147682 | 9.00E-08 |
|  | 7 | 16 | rs2126371 | T | C | 0.317531 | -0.0289453 | 0.00597696 | 1.30E-06 |
|  | 8 | 16 | rs117219913 | C | T | 0.07834 | 0.0476864 | 0.0103813 | 4.40E-06 |
|  | 9 | 20 | rs909570 | A | G | 0.938952 | -0.053135 | 0.0115717 | 4.40E-06 |
| Vitamin C | 1 | 3 | rs7626478 | A | G | 0.720257 | 0.0279796 | 0.00610134 | 4.50E-06 |
|  | 2 | 3 | rs114598078 | T | C | 0.042309 | 0.0655782 | 0.0137636 | 1.90E-06 |
|  | 3 | 3 | rs4481190 | C | A | 0.351041 | -0.0306375 | 0.00574377 | 9.60E-08 |
|  | 4 | 7 | rs74978963 | T | C | 0.008729 | 0.150814 | 0.0310213 | 1.20E-06 |
|  | 5 | 10 | rs61868302 | T | C | 0.060676 | -0.0571013 | 0.0118391 | 1.40E-06 |
|  | 6 | 12 | rs17482258 | T | C | 0.099073 | 0.042829 | 0.00926045 | 3.70E-06 |
|  | 7 | 12 | rs2018201 | G | T | 0.026551 | -0.0808079 | 0.0171646 | 2.50E-06 |
|  | 8 | 13 | rs9540734 | A | G | 0.477524 | -0.0259285 | 0.00548469 | 2.30E-06 |
|  | 9 | 15 | rs4238567 | C | T | 0.522046 | 0.0253068 | 0.00550812 | 4.30E-06 |
|  | 10 | 17 | rs11650824 | A | T | 0.035071 | 0.0794787 | 0.0158822 | 5.60E-07 |
|  | 11 | 22 | rs1883993 | A | G | 0.095419 | 0.044959 | 0.00935324 | 1.50E-06 |
| Vitamin Eα | 1 | 7 | rs1404410 | G | C | 0.2147 | 0.0236 | 0.0052 | 4.57E-06 |
|  | 2 | 7 | rs10245705 | T | C | 0.0181 | -0.0663 | 0.0127 | 1.95E-07 |
|  | 3 | 9 | rs11145330 | C | A | 0.1092 | -0.0324 | 0.0068 | 1.95E-06 |
|  | 4 | 18 | rs7238006 | C | T | 0.0738 | -0.0281 | 0.0057 | 6.77E-07 |
|  | 5 | 22 | rs2074731 | A | C | 0.1654 | -0.0184 | 0.0039 | 2.31E-06 |
| Vitamin Eγ | 1 | 5 | rs10077932 | T | C | 0.1376 | -0.0402 | 0.0087 | 4.08E-06 |
|  | 2 | 9 | rs7038957 | C | T | 0.1688 | 0.0287 | 0.0062 | 3.86E-06 |
|  | 3 | 15 | rs7350776 | G | C | 0.303 | -0.0239 | 0.0052 | 3.86E-06 |
|  | 4 | 15 | rs261301 | C | T | 0.8686 | -0.0323 | 0.0068 | 2.06E-06 |
|  | 5 | 17 | rs1013104 | T | C | 0.4354 | -0.0207 | 0.0045 | 3.83E-06 |
|  | 6 | 19 | rs1060467 | G | A | 0.4097 | -0.0233 | 0.0045 | 2.61E-07 |
|  | 7 | 22 | rs5994305 | G | A | 0.1682 | -0.0307 | 0.0062 | 7.15E-07 |
| Zinc | 1 | 2 | rs10931753 | C | G | NA | -0.129 | 0.028 | 4.94E-06 |
|  | 2 | 4 | rs4333127 | A | G | NA | 0.218 | 0.047 | 3.00E-06 |
|  | 3 | 7 | rs11763353 | G | A | NA | -0.192 | 0.039 | 6.90E-07 |
|  | 4 | 8 | rs1532423 | G | A | NA | -0.178 | 0.026 | 6.40E-12 |
|  | 5 | 11 | rs11232535 | C | T | NA | 0.325 | 0.065 | 6.73E-07 |
|  | 6 | 14 | rs7148590 | A | G | NA | -0.14 | 0.026 | 1.37E-07 |
|  | 7 | 14 | rs10484100 | G | A | NA | -0.209 | 0.045 | 3.30E-06 |
|  | 8 | 15 | rs2120019 | C | T | NA | -0.287 | 0.033 | 1.55E-18 |
| Selenium | 1 | 2 | rs3770549 | T | A | NA | 0.187 | 0.04 | 2.39E-06 |
|  | 2 | 4 | rs6823178 | A | G | NA | -0.125 | 0.026 | 2.32E-06 |
|  | 3 | 5 | rs921943 | T | C | NA | 0.264 | 0.03 | 1.43E-18 |
|  | 4 | 5 | rs11948804 | T | C | NA | 0.236 | 0.047 | 3.90E-07 |
|  | 5 | 16 | rs2631524 | G | A | NA | 0.15 | 0.032 | 1.98E-06 |
|  | 6 | 17 | rs12951643 | A | G | NA | -0.232 | 0.047 | 8.01E-07 |
|  | 7 | 22 | rs9609603 | C | T | NA | 0.123 | 0.027 | 4.00E-06 |
| Carotenoid | 1 | 1 | rs1936052 | T | C | 0.155936 | -0.0361185 | 0.00766833 | 2.50E-06 |
|  | 2 | 1 | rs6660246 | C | A | 0.450266 | -0.0266889 | 0.00553072 | 1.40E-06 |
|  | 3 | 1 | rs12126792 | G | A | 0.011509 | -0.134712 | 0.0280049 | 1.50E-06 |
|  | 4 | 4 | rs77547747 | C | T | 0.056418 | -0.055803 | 0.0118339 | 2.40E-06 |
|  | 5 | 5 | rs6596473 | C | G | 0.299056 | 0.0276423 | 0.00597629 | 3.70E-06 |
|  | 6 | 6 | rs62417408 | G | A | 0.037772 | -0.0691153 | 0.0146553 | 2.40E-06 |
|  | 7 | 8 | rs16898247 | A | G | 0.018968 | -0.107091 | 0.0200309 | 9.00E-08 |
|  | 8 | 9 | rs13295574 | A | G | 0.303383 | -0.0276722 | 0.00598182 | 3.70E-06 |
|  | 9 | 10 | rs3829931 | A | T | 0.973235 | 0.0830829 | 0.0177899 | 3.00E-06 |
|  | 10 | 10 | rs17800766 | C | T | 0.011673 | -0.121685 | 0.0254964 | 1.80E-06 |
|  | 11 | 10 | rs2998143 | G | A | 0.603581 | -0.0278722 | 0.00584016 | 1.80E-06 |
|  | 12 | 13 | rs4771831 | A | G | 0.351886 | -0.0265212 | 0.00576207 | 4.20E-06 |
|  | 13 | 16 | rs116995905 | T | C | 0.009812 | -0.132301 | 0.0288061 | 4.40E-06 |
|  | 14 | 19 | rs366337 | G | A | 0.936764 | 0.0543804 | 0.0112221 | 1.30E-06 |
|  | 15 | 22 | rs117731008 | A | G | 0.016818 | 0.0977133 | 0.0212277 | 4.20E-06 |
|  | 16 | 22 | rs5760695 | C | T | 0.086598 | 0.0468481 | 0.0100556 | 3.20E-06 |

Vitamin Eα is α-tocopherol, and Vitamin Eγ is γ-tocopherol.

**Table S17** Associations between genetically predicted increase in dietary-derived antioxidants and sleep disorder in Mendelian Randomization analyses

| Outcome | Exposure | Method | nsnp | Beta | SE | *P* | Lower CI | Upper CI | OR | Lower CI | Upper CI |
| --- | --- | --- | --- | --- | --- | --- | --- | --- | --- | --- | --- |
| Vitamin A | OSA | MR Egger | 8 | 0.416 | 0.372 | 0.306 | -0.313 | 1.145 | 1.516 | 0.731 | 3.142 |
| Vitamin A | OSA | Weighted median | 8 | -0.053 | 0.211 | 0.802 | -0.466 | 0.360 | 0.949 | 0.628 | 1.434 |
| Vitamin A | OSA | IVW | 8 | -0.092 | 0.155 | 0.554 | -0.396 | 0.212 | 0.912 | 0.673 | 1.237 |
| Vitamin A | OSA | Simple mode | 8 | -0.045 | 0.300 | 0.886 | -0.634 | 0.544 | 0.956 | 0.531 | 1.723 |
| Vitamin A | OSA | Weighted mode | 8 | 0.021 | 0.253 | 0.935 | -0.475 | 0.517 | 1.022 | 0.622 | 1.678 |
| Vitamin C | OSA | MR Egger | 11 | -0.238 | 0.249 | 0.363 | -0.725 | 0.249 | 0.788 | 0.484 | 1.283 |
| Vitamin C | OSA | Weighted median | 11 | -0.077 | 0.158 | 0.625 | -0.387 | 0.233 | 0.926 | 0.679 | 1.262 |
| Vitamin C | OSA | IVW | 11 | -0.062 | 0.121 | 0.607 | -0.299 | 0.174 | 0.940 | 0.742 | 1.191 |
| Vitamin C | OSA | Simple mode | 11 | -0.042 | 0.235 | 0.863 | -0.503 | 0.419 | 0.959 | 0.605 | 1.521 |
| Vitamin C | OSA | Weighted mode | 11 | -0.061 | 0.192 | 0.757 | -0.437 | 0.315 | 0.941 | 0.646 | 1.370 |
| Vitamin Eα | OSA | MR Egger | 5 | -0.639 | 1.731 | 0.736 | -4.033 | 2.754 | 0.528 | 0.018 | 15.707 |
| Vitamin Eα | OSA | Weighted median | 5 | 0.235 | 0.418 | 0.574 | -0.584 | 1.054 | 1.265 | 0.558 | 2.868 |
| Vitamin Eα | OSA | IVW | 5 | -0.065 | 0.459 | 0.888 | -0.964 | 0.834 | 0.937 | 0.382 | 2.303 |
| Vitamin Eα | OSA | Simple mode | 5 | 0.424 | 0.898 | 0.661 | -1.337 | 2.185 | 1.528 | 0.263 | 8.889 |
| Vitamin Eα | OSA | Weighted mode | 5 | 0.575 | 0.537 | 0.344 | -0.477 | 1.627 | 1.778 | 0.621 | 5.090 |
| Vitamin Eγ | OSA | MR Egger | 7 | -1.826 | 0.895 | 0.097 | -3.579 | -0.072 | 0.161 | 0.028 | 0.931 |
| Vitamin Eγ | OSA | Weighted median | 7 | -0.230 | 0.275 | 0.403 | -0.768 | 0.308 | 0.795 | 0.464 | 1.361 |
| Vitamin Eγ | OSA | IVW | 7 | -0.114 | 0.218 | 0.600 | -0.542 | 0.313 | 0.892 | 0.582 | 1.368 |
| Vitamin Eγ | OSA | Simple mode | 7 | -0.347 | 0.444 | 0.464 | -1.218 | 0.524 | 0.707 | 0.296 | 1.688 |
| Vitamin Eγ | OSA | Weighted mode | 7 | -0.396 | 0.376 | 0.333 | -1.132 | 0.341 | 0.673 | 0.322 | 1.406 |
| Zinc | OSA | MR Egger | 7 | -0.027 | 0.115 | 0.825 | -0.253 | 0.199 | 0.974 | 0.777 | 1.221 |
| Zinc | OSA | Weighted median | 7 | -0.044 | 0.037 | 0.233 | -0.115 | 0.028 | 0.957 | 0.891 | 1.029 |
| Zinc | OSA | IVW | 7 | -0.030 | 0.030 | 0.319 | -0.089 | 0.029 | 0.970 | 0.914 | 1.030 |
| Zinc | OSA | Simple mode | 7 | -0.049 | 0.048 | 0.345 | -0.142 | 0.044 | 0.952 | 0.868 | 1.045 |
| Zinc | OSA | Weighted mode | 7 | -0.047 | 0.045 | 0.338 | -0.135 | 0.041 | 0.954 | 0.874 | 1.042 |
| Selenium | OSA | MR Egger | 6 | 0.087 | 0.887 | 0.927 | -1.652 | 1.825 | 1.091 | 0.192 | 6.204 |
| Selenium | OSA | Weighted median | 6 | -0.059 | 0.356 | 0.868 | -0.757 | 0.638 | 0.942 | 0.469 | 1.893 |
| Selenium | OSA | IVW | 6 | -0.094 | 0.290 | 0.746 | -0.662 | 0.474 | 0.910 | 0.516 | 1.606 |
| Selenium | OSA | Simple mode | 6 | -0.491 | 0.529 | 0.396 | -1.529 | 0.546 | 0.612 | 0.217 | 1.727 |
| Selenium | OSA | Weighted mode | 6 | 0.109 | 0.400 | 0.797 | -0.675 | 0.892 | 1.115 | 0.509 | 2.440 |
| Carotene | OSA | MR Egger | 16 | 0.450 | 2.478 | 0.858 | -4.407 | 5.308 | 1.569 | 0.012 | 201.912 |
| Carotene | OSA | Weighted median | 16 | 0.275 | 1.299 | 0.832 | -2.271 | 2.821 | 1.316 | 0.103 | 16.788 |
| Carotene | OSA | IVW | 16 | -0.076 | 1.117 | 0.946 | -2.266 | 2.113 | 0.927 | 0.104 | 8.276 |
| Carotene | OSA | Simple mode | 16 | 0.597 | 1.962 | 0.765 | -3.250 | 4.443 | 1.816 | 0.039 | 85.021 |
| Carotene | OSA | Weighted mode | 16 | 0.521 | 1.590 | 0.747 | -2.594 | 3.637 | 1.685 | 0.075 | 37.977 |
| Vitamin A | Sleep wake | MR Egger | 8 | 1.722 | 3.078 | 0.596 | -4.310 | 7.754 | 5.597 | 0.013 | 2331.814 |
| Vitamin A | Sleep wake | Weighted median | 8 | 0.627 | 1.630 | 0.700 | -2.568 | 3.823 | 1.873 | 0.077 | 45.743 |
| Vitamin A | Sleep wake | IVW | 8 | -0.223 | 1.284 | 0.862 | -2.740 | 2.294 | 0.800 | 0.065 | 9.919 |
| Vitamin A | Sleep wake | Simple mode | 8 | -0.466 | 2.267 | 0.843 | -4.909 | 3.977 | 0.628 | 0.007 | 53.352 |
| Vitamin A | Sleep wake | Weighted mode | 8 | 0.473 | 1.980 | 0.818 | -3.407 | 4.353 | 1.604 | 0.033 | 77.698 |
| Vitamin C | Sleep wake | MR Egger | 11 | -3.935 | 2.020 | 0.083 | -7.896 | 0.025 | 0.020 | 0.000 | 1.025 |
| Vitamin C | Sleep wake | Weighted median | 11 | -3.228 | 1.413 | 0.022 | -5.998 | -0.458 | 0.040 | 0.002 | 0.633 |
| Vitamin C | Sleep wake | IVW | 11 | -3.018 | 0.999 | 0.003 | -4.975 | -1.061 | 0.049 | 0.007 | 0.346 |
| Vitamin C | Sleep wake | Simple mode | 11 | -2.238 | 2.162 | 0.325 | -6.476 | 2.001 | 0.107 | 0.002 | 7.393 |
| Vitamin C | Sleep wake | Weighted mode | 11 | -3.528 | 1.723 | 0.068 | -6.904 | -0.151 | 0.029 | 0.001 | 0.860 |
| Vitamin Eα | Sleep wake | MR Egger | 5 | 12.203 | 8.535 | 0.248 | -4.526 | 28.931 | 199353.168 | 0.011 | 3670406983911.720 |
| Vitamin Eα | Sleep wake | Weighted median | 5 | 4.158 | 3.071 | 0.176 | -1.861 | 10.177 | 63.961 | 0.156 | 26292.656 |
| Vitamin Eα | Sleep wake | IVW | 5 | 0.820 | 2.812 | 0.771 | -4.691 | 6.331 | 2.271 | 0.009 | 561.756 |
| Vitamin Eα | Sleep wake | Simple mode | 5 | 4.439 | 4.735 | 0.402 | -4.842 | 13.719 | 84.669 | 0.008 | 908172.853 |
| Vitamin Eα | Sleep wake | Weighted mode | 5 | 4.565 | 4.277 | 0.346 | -3.818 | 12.948 | 96.082 | 0.022 | 419967.724 |
| Vitamin Eγ | Sleep wake | MR Egger | 7 | -2.403 | 9.633 | 0.813 | -21.283 | 16.477 | 0.090 | 0.000 | 14311848.489 |
| Vitamin Eγ | Sleep wake | Weighted median | 7 | -3.462 | 2.342 | 0.139 | -8.052 | 1.128 | 0.031 | 0.000 | 3.089 |
| Vitamin Eγ | Sleep wake | IVW | 7 | -4.166 | 1.924 | 0.030 | -7.938 | -0.394 | 0.016 | 0.000 | 0.674 |
| Vitamin Eγ | Sleep wake | Simple mode | 7 | -3.532 | 3.479 | 0.349 | -10.351 | 3.288 | 0.029 | 0.000 | 26.776 |
| Vitamin Eγ | Sleep wake | Weighted mode | 7 | -4.221 | 3.512 | 0.275 | -11.105 | 2.664 | 0.015 | 0.000 | 14.349 |
| Zinc | Sleep wake | MR Egger | 7 | 1.015 | 0.958 | 0.338 | -0.862 | 2.892 | 2.759 | 0.422 | 18.026 |
| Zinc | Sleep wake | Weighted median | 7 | -0.067 | 0.300 | 0.824 | -0.654 | 0.521 | 0.936 | 0.520 | 1.683 |
| Zinc | Sleep wake | IVW | 7 | -0.075 | 0.251 | 0.765 | -0.567 | 0.417 | 0.928 | 0.567 | 1.517 |
| Zinc | Sleep wake | Simple mode | 7 | -0.250 | 0.464 | 0.610 | -1.159 | 0.660 | 0.779 | 0.314 | 1.934 |
| Zinc | Sleep wake | Weighted mode | 7 | -0.265 | 0.406 | 0.538 | -1.060 | 0.530 | 0.767 | 0.346 | 1.699 |
| Selenium | Sleep wake | MR Egger | 6 | 0.087 | 0.887 | 0.927 | -1.652 | 1.825 | 1.091 | 0.192 | 6.204 |
| Selenium | Sleep wake | Weighted median | 6 | -0.059 | 0.356 | 0.868 | -0.757 | 0.638 | 0.942 | 0.469 | 1.893 |
| Selenium | Sleep wake | IVW | 6 | -0.094 | 0.290 | 0.746 | -0.662 | 0.474 | 0.910 | 0.516 | 1.606 |
| Selenium | Sleep wake | Simple mode | 6 | -0.491 | 0.529 | 0.396 | -1.529 | 0.546 | 0.612 | 0.217 | 1.727 |
| Selenium | Sleep wake | Weighted mode | 6 | 0.109 | 0.400 | 0.797 | -0.675 | 0.892 | 1.115 | 0.509 | 2.440 |
| Carotene | Sleep wake | MR Egger | 16 | 0.450 | 2.478 | 0.858 | -4.407 | 5.308 | 1.569 | 0.012 | 201.912 |
| Carotene | Sleep wake | Weighted median | 16 | 0.275 | 1.299 | 0.832 | -2.271 | 2.821 | 1.316 | 0.103 | 16.788 |
| Carotene | Sleep wake | IVW | 16 | -0.076 | 1.117 | 0.946 | -2.266 | 2.113 | 0.927 | 0.104 | 8.276 |
| Carotene | Sleep wake | Simple mode | 16 | 0.597 | 1.962 | 0.765 | -3.250 | 4.443 | 1.816 | 0.039 | 85.021 |
| Carotene | Sleep wake | Weighted mode | 16 | 0.521 | 1.590 | 0.747 | -2.594 | 3.637 | 1.685 | 0.075 | 37.977 |

Vitamin Eα is α-tocopherol, and Vitamin Eγ is γ-tocopherol.

**Table S18** Heterogeneity analysis of antioxidants on sleep disorder.

|  |  | Method | MR-Egger intercept | | |
| --- | --- | --- | --- | --- | --- |
| Exposeure | Outcome |  | Q | Q_df | Q_pval |
| Vitamin A | OSA | MR Egger | 2.494 | 6 | 0.869 |
|  |  | IVW | 4.751 | 7 | 0.690 |
| Vitamin C | OSA | MR Egger | 9.286 | 9 | 0.411 |
|  |  | IVW | 9.970 | 10 | 0.443 |
| Vitamin Eα | OSA | MR Egger | 9.030 | 3 | 0.029 |
|  |  | IVW | 9.394 | 4 | 0.052 |
| Vitamin Eγ | OSA | MR Egger | 3.634 | 5 | 0.603 |
|  |  | IVW | 7.473 | 6 | 0.279 |
| Zinc | OSA | MR Egger | 0.910 | 5 | 0.970 |
|  |  | IVW | 0.911 | 6 | 0.989 |
| Selenium | OSA | MR Egger | 5.074 | 4 | 0.280 |
|  |  | IVW | 5.231 | 5 | 0.388 |
| Carotene | OSA | MR Egger | 9.902 | 14 | 0.769 |
|  |  | IVW | 9.912 | 15 | 0.825 |
| VitaminA | Sleep wake | MR Egger | 4.006 | 6 | 0.676 |
|  |  | IVW | 4.490 | 7 | 0.722 |
| VitaminC | Sleep wake | MR Egger | 8.301 | 9 | 0.504 |
|  |  | IVW | 8.574 | 10 | 0.573 |
| VitaminEα | Sleep wake | MR Egger | 3.107 | 3 | 0.375 |
|  |  | IVW | 5.126 | 4 | 0.275 |
| VitaminEγ | Sleep wake | MR Egger | 8.431 | 5 | 0.134 |
|  |  | IVW | 8.490 | 6 | 0.204 |
| Zinc | Sleep wake | MR Egger | 0.690 | 5 | 0.984 |
|  |  | IVW | 2.080 | 6 | 0.912 |
| Selenium | Sleep wake | MR Egger | 2.327 | 4 | 0.676 |
|  |  | IVW | 2.373 | 5 | 0.795 |
| Carotene | Sleep wake | MR Egger | 19.534 | 14 | 0.146 |
|  |  | IVW | 19.615 | 15 | 0.187 |

Vitamin Eα is α-tocopherol, and Vitamin Eγ is γ-tocopherol.

**Table S19** Pleiotropy analysis of antioxidants on sleep disorder.

| Exposeure | Outcome | Egger-intercept | Egger-SE | P-Egger |
| --- | --- | --- | --- | --- |
| VitaminA | OSA | -0.025 | 0.017 | 0.184 |
| VitaminC | OSA | 0.009 | 0.011 | 0.437 |
| VitaminEα | OSA | 0.017 | 0.048 | 0.751 |
| VitaminEγ | OSA | 0.049 | 0.025 | 0.107 |
| Zinc | OSA | -0.001 | 0.024 | 0.977 |
| Selenium | OSA | 0.008 | 0.022 | 0.742 |
| Carotene | OSA | 0.001 | 0.011 | 0.923 |
| VitaminA | Sleep wake | -0.095 | 0.137 | 0.513 |
| VitaminC | Sleep wake | 0.046 | 0.089 | 0.614 |
| VitaminEα | Sleep wake | -0.327 | 0.234 | 0.257 |
| VitaminEγ | Sleep wake | -0.051 | 0.269 | 0.859 |
| Zinc | Sleep wake | -0.234 | 0.199 | 0.291 |
| Selenium | Sleep wake | -0.035 | 0.162 | 0.840 |
| Carotene | Sleep wake | -0.025 | 0.104 | 0.814 |

Vitamin Eα is α-tocopherol, and Vitamin Eγ is γ-tocopherol.
