# Supplementary material for: Antioxidants and the risk of sleep disorders: results from NHANES and two-sample Mendelian randomization study
Source: Front Nutr. 2024 Oct 2;11:1453064. doi: 10.3389/fnut.2024.1453064 (PMC11480095; doi:10.3389/fnut.2024.1453064)
Supplement: Supplementary file 2 [file Data_Sheet_1.docx]

**STROBE-MR checklist of recommended items to address in reports of Mendelian randomization studies**^1^ ^2^

| **Item No.** | **Section** | **Checklist item** | **Page No.** | **Relevant text from manuscript** |
| --- | --- | --- | --- | --- |
| 1 | **TITLE and ABSTRACT** | Indicate Mendelian randomization (MR) as the study’s design in the title and/or the abstract if that is a main purpose of the study |  | Antioxidants and the Risk of Sleep Disorders: Results from NHANES and Two-Sample Mendelian Randomization Study |
|  | **INTRODUCTION** |  |  |  |
| 2 | **Background** | Explain the scientific background and rationale for the reported study. What is the exposure? Is a potential causal relationship between exposure and outcome plausible? Justify why MR is a helpful method to address the study question |  | Even after decades of research, the mechanisms regulating human sleep remain largely unknown. In animal models, sleep deprivation induces cellular oxidative stress, which can be mitigated by antioxidants. Vitamin C has immunomodulatory and antioxidant properties. Vitamin E, a potent antioxidant, can prevent oxidative stress and provides neuroprotective effects for the brain. Current research on how specific antioxidants impact sleep disorders is inconsistent. Notably, meta-analyses show that increasing antioxidant supplements is not always beneficial and can sometimes increase mortality rates. This study aims to evaluate whether CDAI can prevent sleep disorders. |
| 3 | **Objectives** | State specific objectives clearly, including pre-specified causal hypotheses (if any). State that MR is a method that, under specific assumptions, intends to estimate causal effects |  | We used Mendelian randomization (MR) with data from genome-wide association studies (GWAS) on six antioxidants and three sleep disorder subtypes to assess the causal link. This approach aims to establish the potential causal impact of antioxidants on sleep disorders. |
|  | **METHODS** |  |  |  |
| 4 | **Study design and data sources** | Present key elements of the study design early in the article. Consider including a table listing sources of data for all phases of the study. For each data source contributing to the analysis, describe the following: |  | See Table S2 |
|  | a) | Setting: Describe the study design and the underlying population, if possible. Describe the setting, locations, and relevant dates, including periods of recruitment, exposure, follow-up, and data collection, when available. |  | In order to test causal effects of antioxidants on risk of sleep disorders within the MR framework.For antioxidants, we selected vitamin A, C, E, zinc, selenium, and carotene, and for outcomes, we chose two subtypes of sleep disorders: OSA and sleep wake disturbances. Specific details can be found in the supplementary materials Table S2. |
|  | b) | Participants: Give the eligibility criteria, and the sources and methods of selection of participants. Report the sample size, and whether any power or sample size calculations were carried out prior to the main analysis |  | See answer to the point above |
|  | c) | Describe measurement, quality control and selection of genetic variants |  | The research is based on three hypotheses: (1) The instrumental variable is strongly correlated with diet-derived antioxidants; (2) The instrumental variable is not correlated with the confounding factors; (3) The instrumental variable is not directly related to sleep disorders, and its effect on sleep disorders can only be through diet-derived antioxidants. This is ensured by using SNPs associated with vitamin A, C, E, zinc, selenium, and carotene at a genome-wide significance level (p-value < 5 x 10^-6). We also calculated the F-statistic, with F > 10 serving as a metric of the strength of our MR instruments. |
|  | d) | For each exposure, outcome, and other relevant variables, describe methods of assessment and diagnostic criteria for diseases |  | Descriptives of the GWAS populations can be found in the respective GWAS publications |
|  | e) | Provide details of ethics committee approval and participant informed consent, if relevant |  | Not applicable. |
| 5 | **Assumptions** | Explicitly state the three core IV assumptions for the main analysis (relevance, independence and exclusion restriction) as well assumptions for any additional or sensitivity analysis |  | The research is based on three hypotheses: (1) The instrumental variable is strongly correlated with diet-derived antioxidants; (2) The instrumental variable is not correlated with the confounding factors; (3) The instrumental variable is not directly related to sleep disorders, and its effect on sleep disorders can only be through diet-derived antioxidants. This is ensured by using SNPs associated with vitamin A, C, E, zinc, selenium, and carotene at a genome-wide significance level (p-value < 5 x 10^-6). We also calculated the F-statistic, with F > 10 serving as a metric of the strength of our MR instruments. |
| 6 | **Statistical methods: main analysis** | Describe statistical methods and statistics used |  |  |
|  | a) | Describe how quantitative variables were handled in the analyses (i.e., scale, units, model) |  | We computed the power in our main MR analyses. The handling of quantitative variables in the analyses was as follows: We used the complete set of antioxidant SNPs as instrumental variables to assess the causal effect of antioxidant levels on the risk of two subtypes of sleep disorders (OSA and sleep wake disturbances). Antioxidant levels were measured using established assays, considering the variance explained by their genetic instruments. For the power calculation, we used an established MR power calculation method, setting the significance level at 0.05. We calculated the MR odds ratio (OR) for sleep disorders to quantify the effect, ensuring the robustness of the statistical analysis. |
|  | b) | Describe how genetic variants were handled in the analyses and, if applicable, how their weights were selected |  | The TwoSampleMR R package, with its default parameters (LD distance > 10,000 kb, r2 < 0.001), was used to select antioxidant SNPs, harmonize them between the exposure and outcome GWAS, and calculate the various MR estimates (IVW, weighted median, MR-Egger, and weighted mode) of our main and sensitivity analyses. Random effects IVW was used, given the evidence of heterogeneity in our main analyses. Scatter plots and forest plots to visualize the MR estimates were generated using the TwoSampleMR R package. Our MR-PRESSO analyses were performed using the MR-PRESSO R package. |
|  | c) | Describe the MR estimator (e.g. two-stage least squares, Wald ratio) and related statistics. Detail the included covariates and, in case of two-sample MR, whether the same covariate set was used for adjustment in the two samples |  | Specifically, we used the PhenoScanner database to filter the MR instruments for antioxidants, excluding SNPs associated with confounding factors related to the antioxidants-sleep disorders association. This approach has been detailed in previous MR studies. We conducted sensitivity MR analyses, excluding antioxidant SNPs associated with confounders at a genome-wide level. |
|  | d) | Explain how missing data were addressed |  | If there were missing data, for antioxidant-related SNPs not directly present in the GWAS, we would have selected proxy SNPs (LD r^2 > 0.8) using the LDproxy function in ldlink in matching populations from the 1000 Genomes Phase 3 panel. However, there were no missing data in this study. |
|  | e) | If applicable, indicate how multiple testing was addressed |  | Not applicable |
| 7 | **Assessment of assumptions** | Describe any methods or prior knowledge used to assess the assumptions or justify their validity |  | In order to conduct any MR study, the SNPs used as instrumental variables (IVs) of an exposure must satisfy three main assumptions. The first assumption (relevance assumption) requires that these SNPs should be strongly associated with the exposure, in this case, the antioxidants vitamin A, C, E, zinc, selenium, and carotene. This is ensured by using SNPs associated with these antioxidants at a genome-wide significance level (p-value <5 x 10^-6). We also calculated the F-statistic for our set of antioxidant SNPs, with F > 10 serving as a metric of the strength of our MR instruments.  The second assumption (independence assumption) requires that the SNPs used as MR instruments should not be associated with confounders of the association between the exposure and outcome. We undertook multiple sensitivity analyses with different sets of antioxidant SNPs to ensure that our MR results are not biased by confounders. Additionally, we performed a multivariable MR analysis considering both antioxidants and BMI as exposures.  The third MR assumption (exclusion restriction assumption) requires that the SNP-IVs are associated with the outcome (here, two types of sleep disorders) solely via the exposure (here, antioxidant levels). Pleiotropy refers to a scenario where this assumption is violated. In order to test for the presence of pleiotropy, we conducted sensitivity analyses applying various pleiotropy-robust MR methods, each with its own assumptions. |
| 8 | **Sensitivity analyses and additional analyses** | Describe any sensitivity analyses or additional analyses performed (e.g. comparison of effect estimates from different approaches, independent replication, bias analytic techniques, validation of instruments, simulations) |  | See Supplementary Materials Table S17-S19 |
| 9 | **Software and pre-registration** |  |  |  |
|  | a) | Name statistical software and package(s), including version and settings used |  | We used the TwoSampleMR R package (version 0.5.6), with its default parameters (LD distance > 10,000 kb, r2 < 0.001), to select antioxidant SNPs (vitamin A, C, E, zinc, selenium, and carotene), harmonize them between the exposure and outcome GWAS, and calculate the various MR estimates (IVW, weighted median, MR-Egger, and weighted mode) for our main and sensitivity analyses. Random effects IVW was used, given the evidence of heterogeneity in our main analyses. Scatter plots and forest plots to visualize the MR estimates were generated using the TwoSampleMR R package. We used the MVMR R package for our multivariable MR analysis . Our MR-PRESSO analyses were performed using the MR-PRESSO R package . |
|  | b) | State whether the study protocol and details were pre-registered (as well as when and where) |  | Not applicable |
|  | **RESULTS** |  |  |  |
| 10 | **Descriptive data** |  |  |  |
|  | a) | Report the numbers of individuals at each stage of included studies and reasons for exclusion. Consider use of a flow diagram |  | See Figure S2 for supplementary material |
|  | b) | Report summary statistics for phenotypic exposure(s), outcome(s), and other relevant variables (e.g. means, SDs, proportions) |  | See Table S2 for supplementary material |
|  | c) | If the data sources include meta-analyses of previous studies, provide the assessments of heterogeneity across these studies |  | See answer to point above |
|  | d) | For two-sample MR:  i.  Provide justification of the similarity of the genetic variant-exposure associations between the exposure and outcome samples  ii.  Provide information on the number of individuals who overlap between the exposure and outcome studies |  | To our knowledge, there is no overlap between  exposure data and outcome data |
| 11 | **Main results** |  |  |  |
|  | a) | Report the associations between genetic variant and exposure, and between genetic variant and outcome, preferably on an interpretable scale |  | As shown in Table 2, MR analysis demonstrated that genetically determined selenium reduces the risk of OSA (OR = 0.992, 95% CI 0.860–0.989, P = 0.023). Furthermore, vitamin E (γ-tocopherol) and vitamin C were protective against sleep-wake disorders, with OR = 0.016 (95% CI 0.001–0.674, P = 0.03) and OR = 0.049 (95% CI 0.007–0.346, P = 0.002), respectively. |
|  | b) | Report MR estimates of the relationship between exposure and outcome, and the measures of uncertainty from the MR analysis, on an interpretable scale, such as odds ratio or relative risk per SD difference |  | See point above |
|  | c) | If relevant, consider translating estimates of relative risk into absolute risk for a meaningful time period |  | Not applicable |
|  | d) | Consider plots to visualize results (e.g. forest plot, scatterplot of associations between genetic variants and outcome versus between genetic variants and exposure) |  | See Supplemental Figure S3-S5 |
| 12 | **Assessment of assumptions** |  |  |  |
|  | a) | Report the assessment of the validity of the assumptions |  | In the MR analyses, potential SNP heterogeneity was detected only in the MR-Egger analysis of Vitamin E (α-tocopherol) and OSA (P = 0.029). No evidence of horizontal pleiotropy was detected. All antioxidant SNPs used in the MR analyses had an F-statistic greater than 10. |
|  | b) | Report any additional statistics (e.g., assessments of heterogeneity across genetic variants, such as *I^2^*, Q statistic or E-value) |  | See Table S18 of the supplementary material |
| 13 | **Sensitivity analyses and additional analyses** |  |  |  |
|  | a) | Report any sensitivity analyses to assess the robustness of the main results to violations of the assumptions |  | See Table S18-S19 of the supplementary material |
|  | b) | Report results from other sensitivity analyses or additional analyses |  | See Table S18-S19 of the supplementary material |
|  | c) | Report any assessment of direction of causal relationship (e.g., bidirectional MR) |  | One-way MR |
|  | d) | When relevant, report and compare with estimates from non-MR analyses |  | Not applicable |
|  | e) | Consider additional plots to visualize results (e.g., leave-one-out analyses) |  | Not applicable |
|  | **DISCUSSION** |  |  |  |
| 14 | **Key results** | Summarize key results with reference to study objectives |  | Third paragraph of the Discussion |
| 15 | **Limitations** | Discuss limitations of the study, taking into account the validity of the IV assumptions, other sources of potential bias, and imprecision. Discuss both direction and magnitude of any potential bias and any efforts to address them |  | See Strengths and Limitations selection |
| 16 | **Interpretation** |  |  |  |
|  | a) | Meaning: Give a cautious overall interpretation of results in the context of their limitations and in comparison with other studies |  | The MR study was conducted in a European population, and whether the results of this study are applicable to other populations is to be studied |
|  | b) | Mechanism: Discuss underlying biological mechanisms that could drive a potential causal relationship between the investigated exposure and the outcome, and whether the gene-environment equivalence assumption is reasonable. Use causal language carefully, clarifying that IV estimates may provide causal effects only under certain assumptions |  | See Discussion section |
|  | c) | Clinical relevance: Discuss whether the results have clinical or public policy relevance, and to what extent they inform effect sizes of possible interventions |  | See Discussion section |
| 17 | **Generalizability** | Discuss the generalizability of the study results (a) to other populations, (b) across other exposure periods/timings, and (c) across other levels of exposure |  | See paragraph on limitations |
|  | **OTHER INFORMATION** |  |  |  |
| 18 | **Funding** | Describe sources of funding and the role of funders in the present study and, if applicable, sources of funding for the databases and original study or studies on which the present study is based |  | See relative section |
| 19 | **Data and data sharing** | Provide the data used to perform all analyses or report where and how the data can be accessed, and reference these sources in the article. Provide the statistical code needed to reproduce the results in the article, or report whether the code is publicly accessible and if so, where |  | R scripts used to generate the results of this study are available upon request to the corresponding author. Summary-level results of all GWAS used in this study are publicly available through GWAS catalog. |
| 20 | **Conflicts of Interest** | All authors should declare all potential conflicts of interest |  | The authors declare no conflict of interest. |

This checklist is copyrighted by the Equator Network under the Creative Commons Attribution 3.0 Unported (CC BY 3.0) license.

1. Skrivankova VW, Richmond RC, Woolf BAR, Yarmolinsky J, Davies NM, Swanson SA, et al. Strengthening the Reporting of Observational Studies in Epidemiology using Mendelian Randomization (STROBE-MR) Statement. JAMA. 2021;under review.

2. Skrivankova VW, Richmond RC, Woolf BAR, Davies NM, Swanson SA, VanderWeele TJ, et al. Strengthening the Reporting of Observational Studies in Epidemiology using Mendelian Randomisation (STROBE-MR): Explanation and Elaboration. BMJ. 2021;375:n2233.

|  |  |
| --- | --- |
