# Supplementary material for: Antioxidants and the risk of sleep disorders: results from NHANES and two-sample Mendelian randomization study
Source: Front Nutr. 2024 Oct 2;11:1453064. doi: 10.3389/fnut.2024.1453064 (PMC11480095; doi:10.3389/fnut.2024.1453064)
Supplement: Supplementary file 3 [file Image_1.pdf]

Supplementary Figures for

Antioxidants and the Risk of Sleep Disorders: Results from NHANES and Two-Sample Mendelian Randomization Study

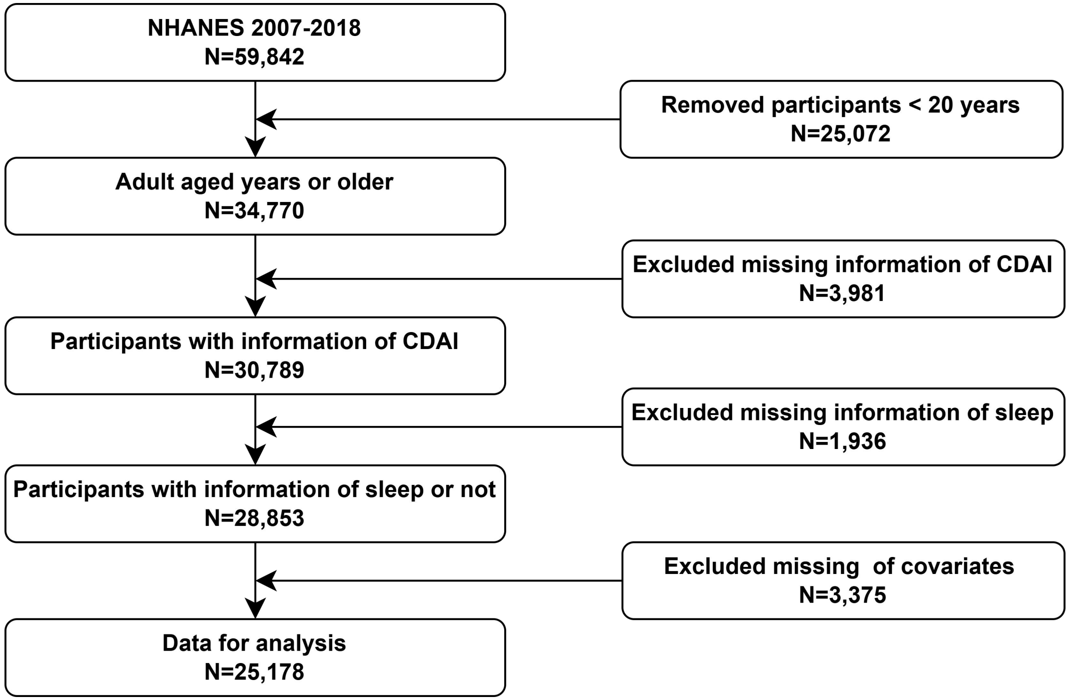

Figure S1 Flow chart of eligible National Health and Nutrition Examination Survey (NHANES) participants included in this study.

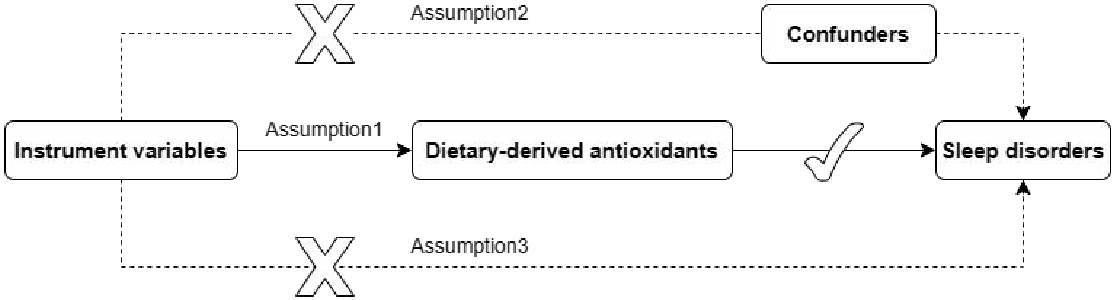

Figure S2 The research is based on three hypotheses: (1) The instrumental variable is strongly correlated with diet-derived antioxidants; (2) The

instrumental variable is not correlated with the confounding factors; (3) The instrumental variable is not directly related to sleep disorders, and its effect on sleep disorders can only be through diet-derived antioxidants to reflect.

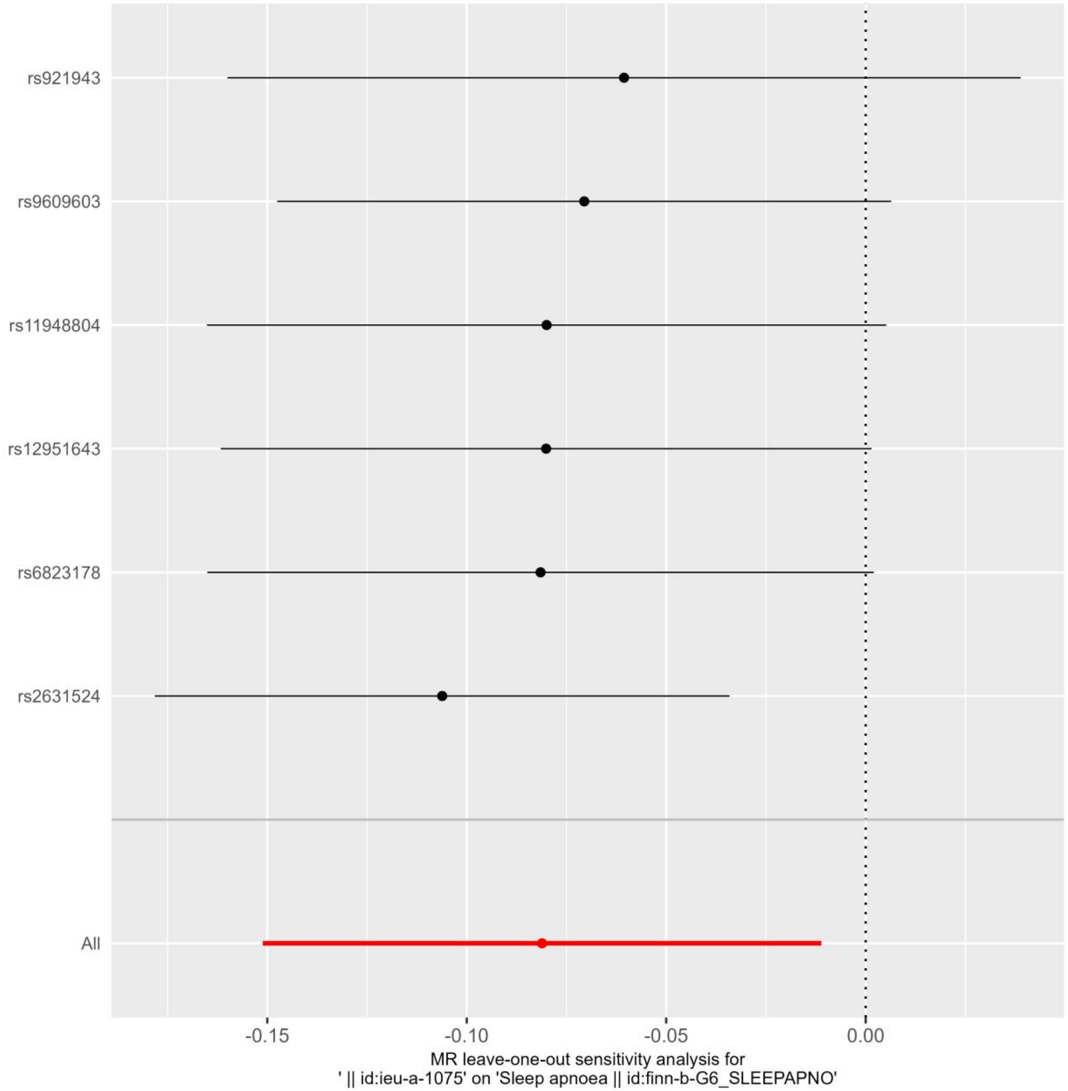

Figure S3 Results of leave-one-out sensitivity analysis for Selenium on OSA.

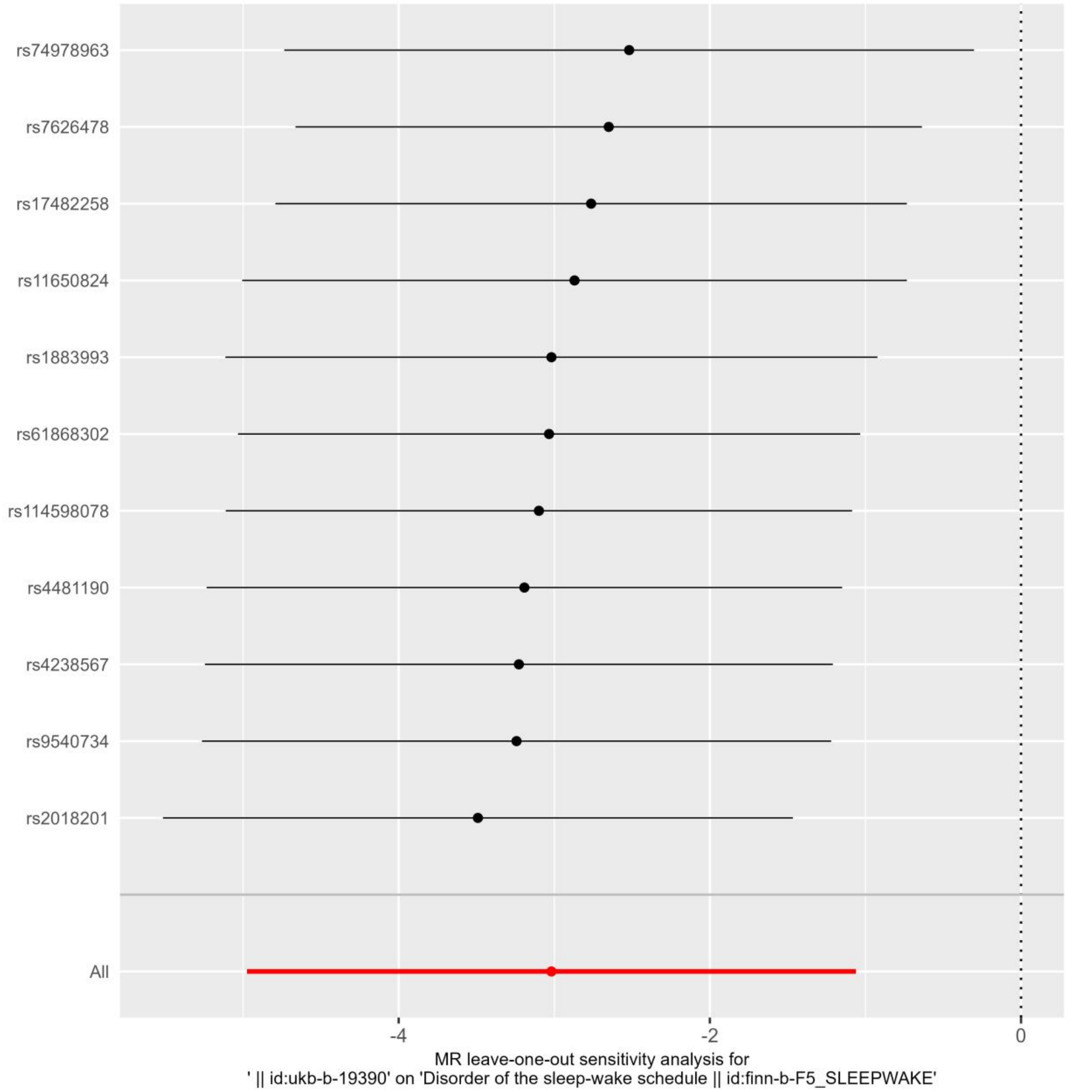

Figure S4 Results of leave-one-out sensitivity analysis for Vitamin C on Sleep wake.

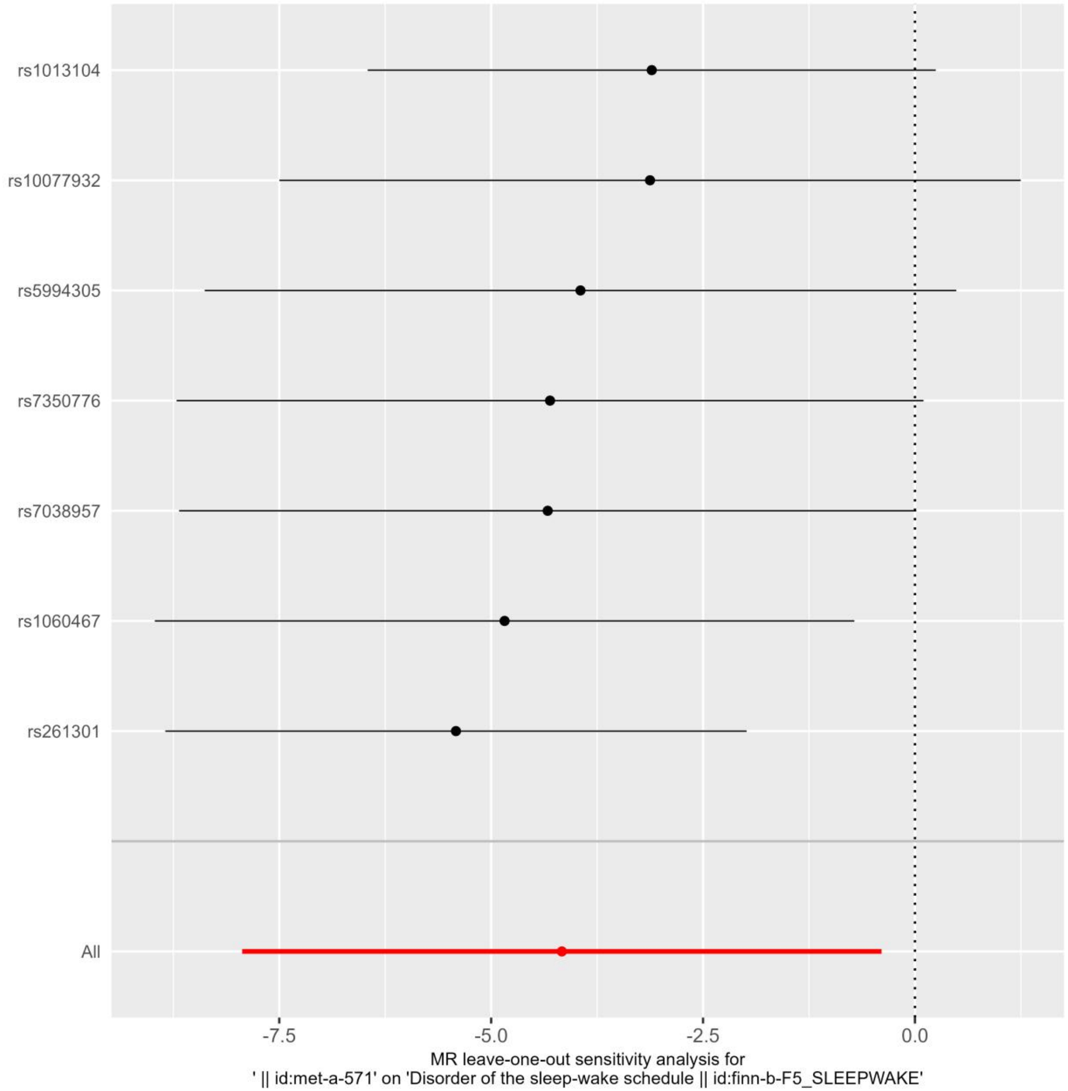

Figure S5 Results of leave-one-out sensitivity analysis for Vitamin E (γ-tocopherol) on Sleep wake.

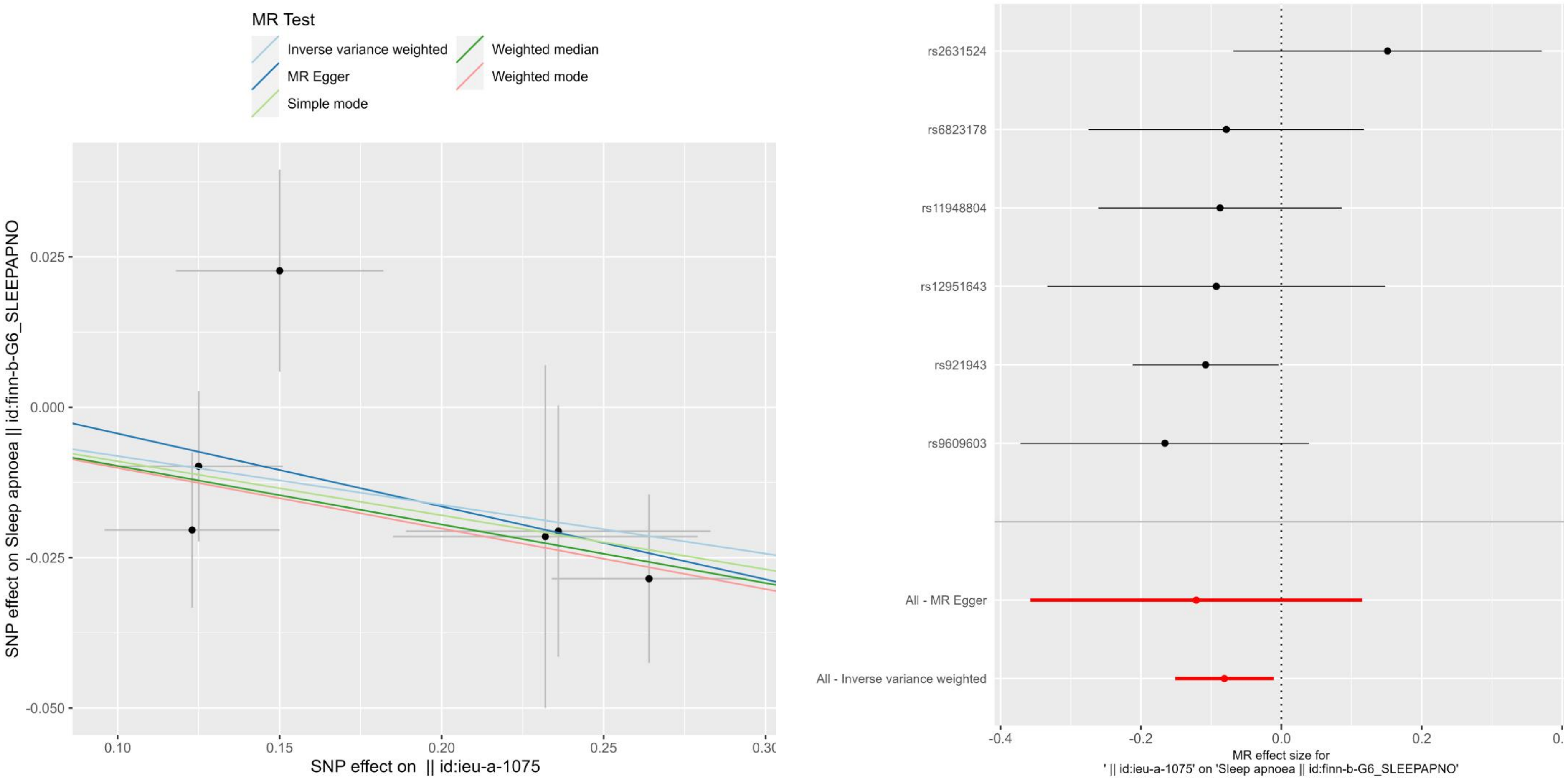

Figure S6 Scatter plot and forest plot of the main MR analysis with selenium and OSA.

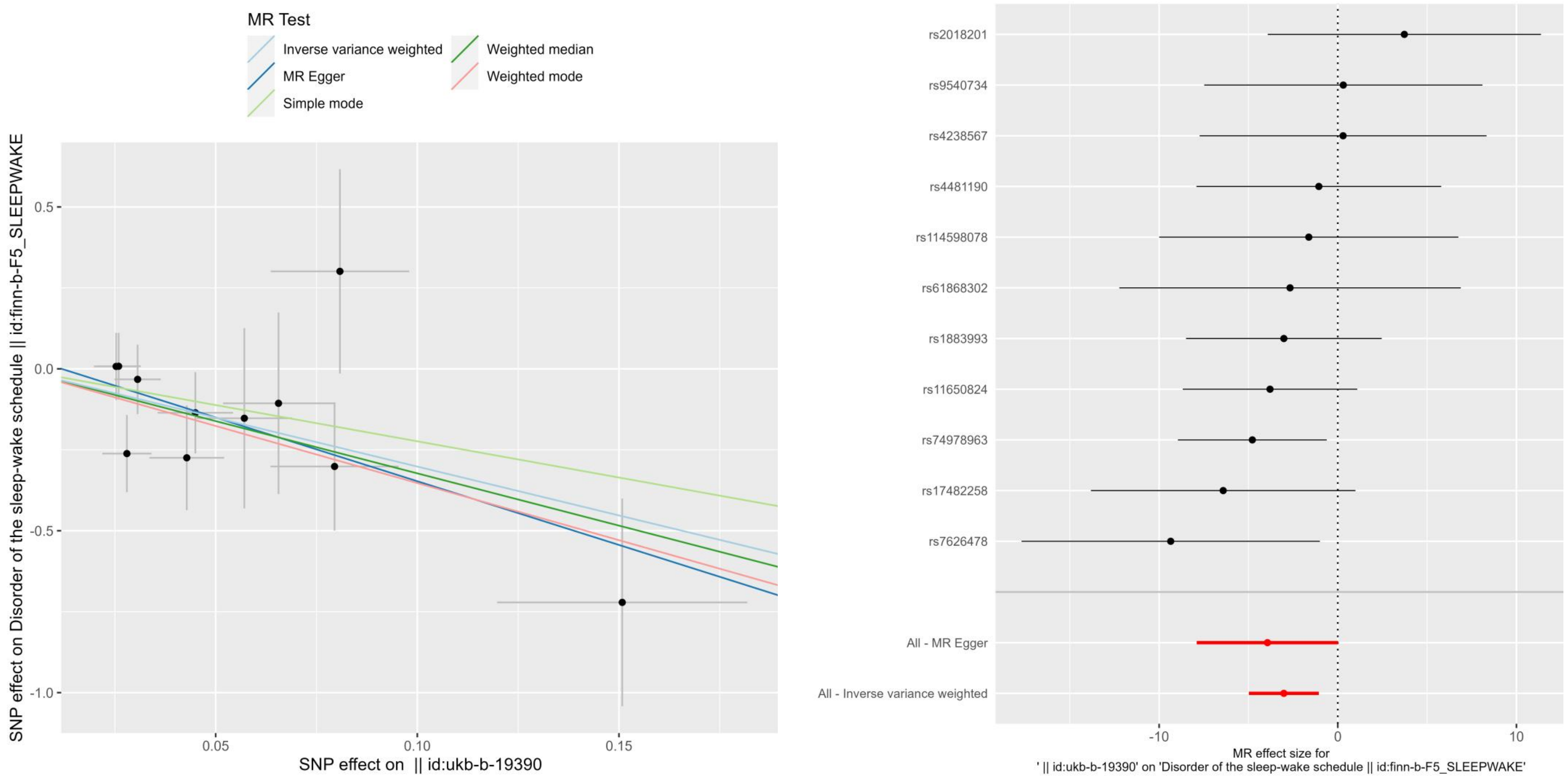

Figure S7 Scatter plot and forest plot of the main MR analysis with Vitamin C and Sleep wake.

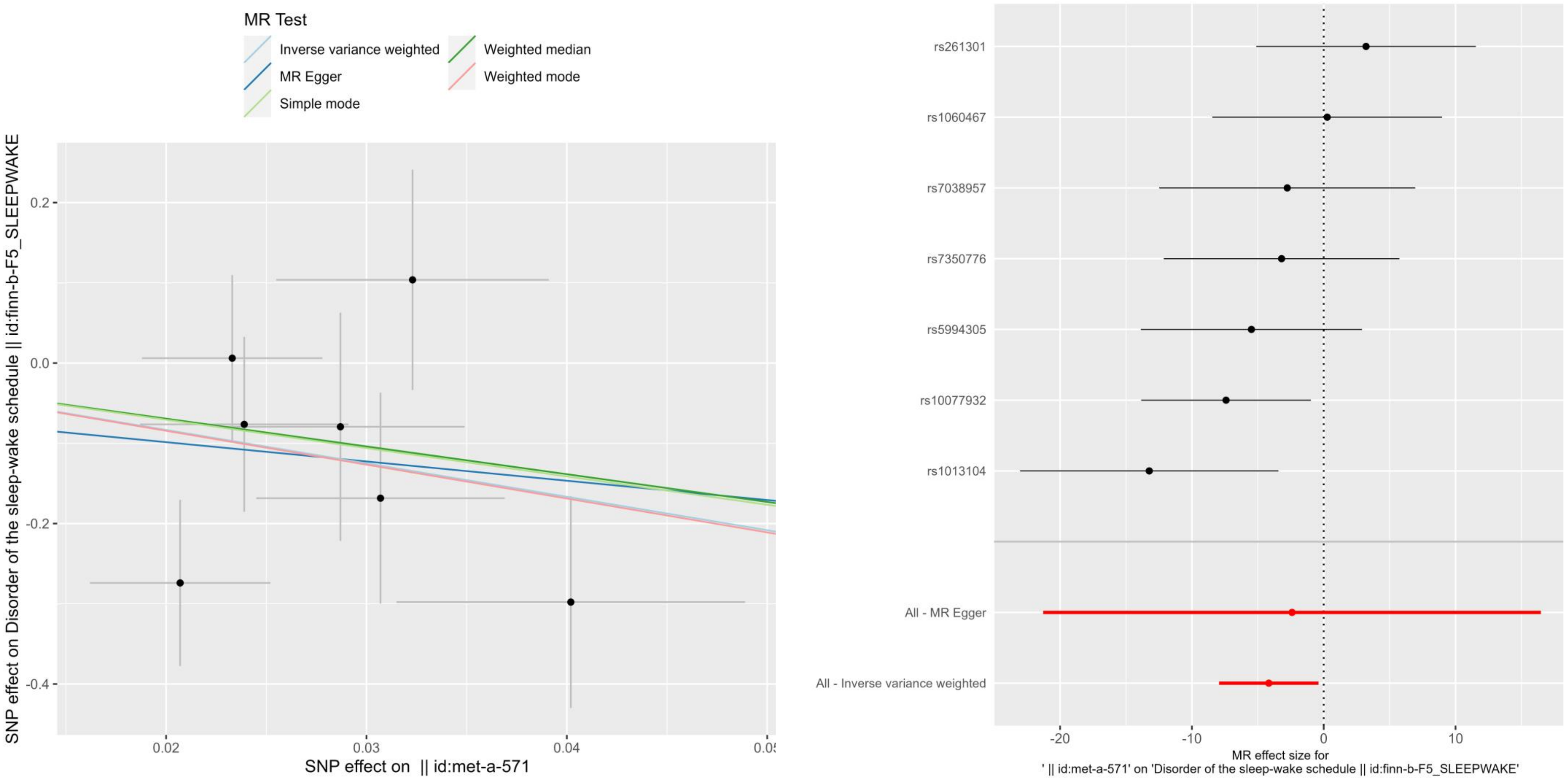

Figure S8 Scatter plot and forest plot of the main MR analysis with Vitamin E (γ -tocopherol) and Sleep wake.
